# Transposable Elements *versus* the Fungal Genome: Impact on Whole-Genome Architecture and Transcriptional Profiles

**DOI:** 10.1371/journal.pgen.1006108

**Published:** 2016-06-13

**Authors:** Raúl Castanera, Leticia López-Varas, Alessandra Borgognone, Kurt LaButti, Alla Lapidus, Jeremy Schmutz, Jane Grimwood, Gúmer Pérez, Antonio G. Pisabarro, Igor V. Grigoriev, Jason E. Stajich, Lucía Ramírez

**Affiliations:** 1 Genetics and Microbiology Research Group, Department of Agrarian Production, Public University of Navarre, Pamplona, Navarre, Spain; 2 U.S. Department of Energy Joint Genome Institute, Walnut Creek, California, United States of America; 3 Center for Algorithmic Biotechnology, St. Petersburg State University, St. Petersburg, Russia; 4 Hudson Alpha Institute for Biotechnology, Huntsville, Alabama, United States of America; 5 Department of Plant Pathology and Microbiology, Institute for Integrative Genome Biology, University of California-Riverside, Riverside, California, United States of America; University of Utah School of Medicine, UNITED STATES

## Abstract

Transposable elements (TEs) are exceptional contributors to eukaryotic genome diversity. Their ubiquitous presence impacts the genomes of nearly all species and mediates genome evolution by causing mutations and chromosomal rearrangements and by modulating gene expression. We performed an exhaustive analysis of the TE content in 18 fungal genomes, including strains of the same species and species of the same genera. Our results depicted a scenario of exceptional variability, with species having 0.02 to 29.8% of their genome consisting of transposable elements. A detailed analysis performed on two strains of *Pleurotus ostreatus* uncovered a genome that is populated mainly by Class I elements, especially LTR-retrotransposons amplified in recent bursts from 0 to 2 million years (My) ago. The preferential accumulation of TEs in clusters led to the presence of genomic regions that lacked intra- and inter-specific conservation. In addition, we investigated the effect of TE insertions on the expression of their nearby upstream and downstream genes. Our results showed that an important number of genes under TE influence are significantly repressed, with stronger repression when genes are localized within transposon clusters. Our transcriptional analysis performed in four additional fungal models revealed that this TE-mediated silencing was present only in species with active cytosine methylation machinery. We hypothesize that this phenomenon is related to epigenetic defense mechanisms that are aimed to suppress TE expression and control their proliferation.

## Author Summary

Transposable elements (TEs) are enigmatic genetic units that have played important roles in the evolution of eukaryotic genomes. Since their discovery in the 1950s, they have gained increasing attention and are known today as active genome modelers in multiple species. Although these elements have been widely studied in plants, much less is known about their occurrence and impact on the fungal kingdom. Using a diverse set of basidiomycete and ascomycete fungi, we quantified and characterized a huge diversity of DNA and RNA transposable elements, and we identified species that had 0.02 to 29.8% of their genomes occupied by transposable elements. In addition, using our basidiomycete model *Pleurotus ostreatus*, we demonstrated how TE insertions produced detrimental effects on the expression of upstream and downstream genes, which were downregulated compared with the control groups. This silencing mechanism was present in the basidiomycetes tested but exhibited a patchy distribution in ascomycetes, and might be related to specific genome defense mechanisms that control transposon proliferation. This finding reveals the broader impact of transposable elements in fungi. In addition to their importance as long-term evolutionary forces, they play major roles in the more dynamic transcriptome regulation of certain species.

## Introduction

Transposable elements (TEs) are mobile genetic units that colonize prokaryotic and eukaryotic genomes and generate intra- and inter-specific variability. Despite the ubiquity of TEs in the eukaryotic domain, the genome fraction occupied by these elements is highly diverse, accounting for approximately 3% in yeast genomes [1], up to 50% in mammalian genomes [2], and more than 80% in some plants, including wheat or maize [3,4]. The expansion of these elements is mediated by transposition events that can lead to their own duplication. TEs are classified into two classes based on transposition mechanisms. Class I elements transpose via RNA intermediates and include five orders (LTR, DIRS, PLE, LINE, and SINE) that are differentiated based on their structure and transposition system [5,6]. Class II encompasses elements that transpose directly from DNA to DNA. This class is divided into two subclasses. One includes the TIR and Crypton orders, and the other contains Helitrons and Mavericks [5]. The majority of transposable elements generate target site duplications at their insertion sites (TSD), which are formed as part of the insertion process. Exceptions include Helitrons [7] and the recently discovered Spy elements [8]. In addition, TE families are formed by both autonomous (coding for the proteins necessary for its transposition) and non-autonomous elements that rely on compatible transposases/retrotransposases for their mobilization.

Transposable elements can be considered selfish elements that parasitize their host genomes, and eukaryotes have developed defense mechanisms for preventing their expansion. Three mechanisms of TE silencing have been described in fungi: i) repeat-induced point mutations (RIP) [9], ii) transposon methylation [10,11], and iii) RNA-mediated gene silencing (quelling and meiotic silencing) [12,13]. Repeat-induced point mutations were originally described in *Neurospora crassa* and have been more recently studied in a broad range of filamentous fungi [14–16]. Transposon DNA methylation has been increasingly studied in the last few years, and recent genome-wide methylation analyses confirm the importance of this epigenetic mechanism in the control of TE proliferation in fungi [11,17,18]. Quelling and meiotic silencing occur through the detection of aberrant RNAs, which trigger RNAi pathway genes to silence. Meiotic silencing occurs when chromosomal regions are unpaired during meiosis, such as when a TE is present in one parent but not in the other. Previous studies have shown that meiotic silencing targets unpaired transposable elements [19].

Although TEs were originally considered “junk DNA”, we know today that the activity of these elements has strong consequences for genome architecture and that they are key drivers in rapid shifts in eukaryotic genome size [6,20]. Due to their repetitive nature, TEs promote chromosomal rearrangements through homologous recombination and alternative transposition [21]. TE activity can also shape genome function in multiple ways. Transposition events can lead to insertional mutations [22], which can modify or disrupt gene expression, as well as generate new proteins by exon shuffling and TE domestication [23,24]. In addition, TEs are powerful sources of regulatory sequences [25] that can be spread across the genome, rewiring pre-established networks or even creating new ones [26]. Transposable elements are associated with several classes of small RNAs that regulate the expression of multiple genes at the post-transcriptional level [27]. These reasons, among others, have transformed the originally underestimated importance of TEs into a new, exciting subject of study. This is especially relevant in fungi because international sequencing efforts are rapidly increasing the availability of genome sequences of divergent species with different lifestyles [28,29].

Fungal genomes are generally smaller than those of plants and animals, which greatly facilitates their assembly and annotation. However, the accurate annotation and quantification of transposable elements in a genome are not simple tasks, especially in draft assemblies with many scaffolds. Factors such the divergence between TE copies (due to mutations and rearrangements) or the occurrence of nested elements complicate the annotation process and necessitate the use of different algorithms to achieve reliable results [30,31]. With the rapid generation of fungal genomes, TE annotation has typically been performed using different strategies, thus limiting the ability to draw robust conclusions about the differences in TE family expansion in different species when copy differences can be ascribed to either methodological differences or biological variation. Recent comprehensive analyses of fungal TEs have described an exceptional variability in the repeat content [15,28,29], in which amplification events tend to be more related to the fungal lifestyle than to phylogenetic proximity [15,32]. LTR-retrotransposons are usually the most abundant mobile elements in fungal genomes, especially those that belong to the Gypsy and Copia superfamilies. In contrast, DNA elements generally constitute a smaller fraction of the fungal repeats, although in some species such as *Fusarium oxysporum*, they have undergone important amplifications in lineage-specific genomic regions [33].

In this study, we used a multi-approach pipeline for TE annotation in a collection of fungal genomes of varying phylogenetic distances and a detailed analysis of TEs in two strains of *P*. *ostreatus*. This species is a white rot basidiomycete fungus that grows on tree stumps in its natural environment. Its life cycle alternates between monokaryotic (haploid) and dikaryotic (dihaploid) mycelial phases. When two compatible monokaryotic hyphae fuse, a dikaryotic mycelium forms that is able to perform karyogamy, which occurs at the end of the life cycle, immediately before the onset of meiosis. Our results depict a *P*. *ostreatus* TE landscape dominated by Class I elements that tend to aggregate in non-homologous clusters. These clusters have profound impacts on the genome architecture at intra and inter-specific levels. In addition, we show that TE insertions modulate the global transcriptome of *P*. *ostreatus* and other fungi.

## Results

### Status of *P*. *ostreatus* PC15 and PC9 genome assemblies

The two monokaryotic strains of *P*. *ostreatus* used in this study were sequenced by the Joint Genome Institute (JGI). PC15 was sequenced with the Sanger whole-genome shotgun approach [34], and PC9 was sequenced using Sanger whole genome shotgun and 454 paired end sequencing reads. PC15 genome assembly version 2.0 (34.3 Mb) was subjected to targeted genome improvement which led to a complete assembly of 12 scaffolds with a very low gap content (1 gap of 91 base pairs in the whole assembly) that matched the corresponding *P*. *ostreatus* chromosomes (eleven nuclear plus one mitochondrial chromosome) [35]. In contrast, PC9 assembly v1.0 (35.6 Mb) contains 572 scaffolds and a total of 476 gaps that cover 9.72% of the whole assembly.

### TE content in *P*. *ostreatus*

**Fig 1:**
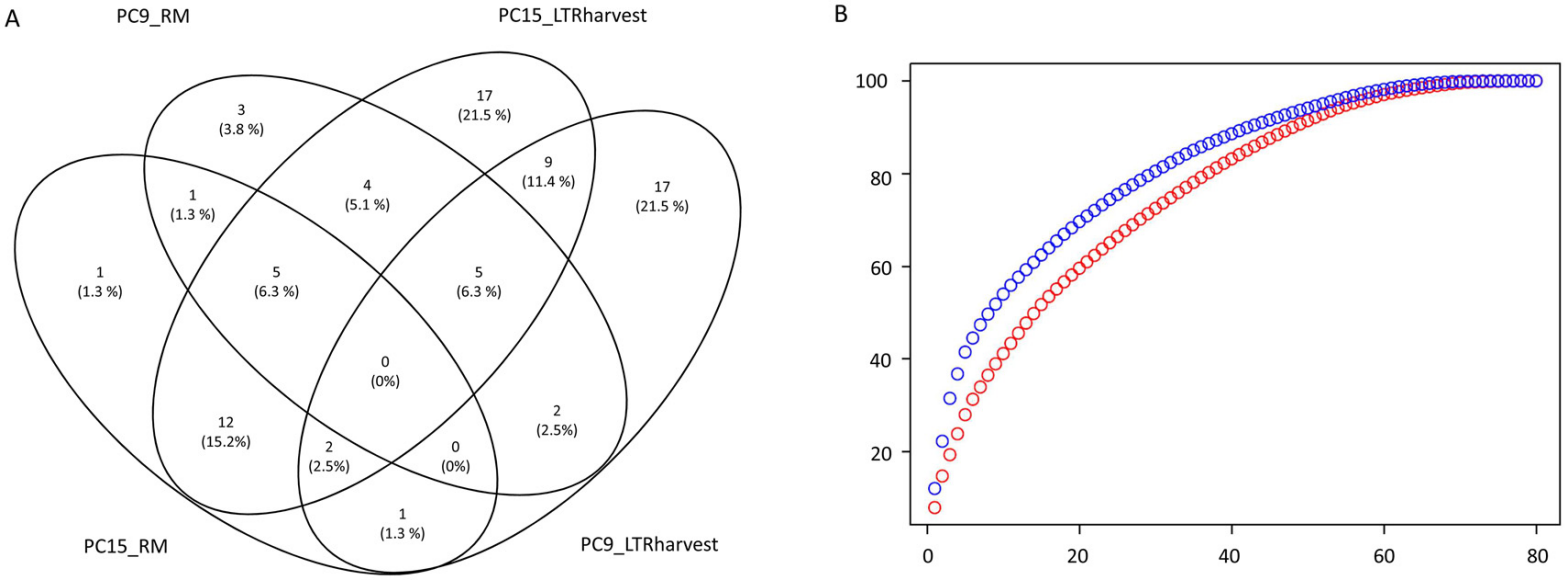
Detection and composition of *P*. *ostreatus* TE families. Venn diagram showing the number of TE families and their percentage of the total library (in parenthesis) identified in PC15 and PC9 genomes by RepeatModeler (RM) and LTRharvest (A). Cumulative plot showing the number of TE families *vs* total TE fraction (B). PC15 is shown in blue and PC9 in red.

Two monokaryotic strains of the basidiomycete *P*. *ostreatus* (PC9 and PC15) [34, 35] were used as a model to analyze differences in the occurrence and expansion of transposable element families. We identified and classified 80 TE families based on structural features and homology to previously described elements (Table 1). These families accounted for 6.2 and 2.5% of the total genome size in PC15 and PC9 genomes, respectively. In addition, we found 144 repeat-like consensus sequences that could not be reliably classified and occupied 3.6 and 2.3% of PC15 and PC9 assemblies, respectively. These elements are referred to hereafter as ‘unknown’ (S1 Table), and were not used in downstream analyses. Our integrated pipeline combined *de novo* predictions of LTRharvest [36] and RepeatModeler (http://www.repeatmasker.org), which were run on the two *P*. *ostreatus* genomes and merged to obtain a final TE library. This library was used then by RepeatMasker (http://www.repeatmasker.org) to detect and mask TE copies in each genome assembly. Our results showed that the merging strategy clearly outperformed the four independent approaches in terms of the number of detected families (Fig 1A). In fact, none of the TE families could be simultaneously detected by all four approaches, and very few were detected by three. In addition, up to 38 families (48% of the total) were detected by only one of the four methods. The distribution of family sizes showed that 9 of the 80 families accounted for the N50 repeat fraction in PC15 (50% of the total TE sequences), whereas 15 families accounted for the N50 repeat fraction in PC9 (Fig 1B).

**Table 1:** Summary of detected TE families in *P*. *ostreatus* strains PC15 and PC9.

Family	Classification	Length (kb)	PC15		PC9	
			Copies *	Kb	Copies *	Kb
Copia_1	LTR-retrotransposon/Copia	4.1	17 (7)	48.2	4 (0)	1.9
Copia_2	LTR-retrotransposon/Copia	5.4	19 (5)	36.1	10 (1)	6.8
Copia_3	LTR-retrotransposon/Copia	6.0	32 (2)	27.9	15 (0)	3.9
Copia_4	LTR-retrotransposon/Copia	5.5	17 (1)	24.2	6 (0)	2.0
Copia_5	LTR-retrotransposon/Copia	6.6	8 (3)	20.6	9 (0)	9.8
Copia_6	LTR-retrotransposon/Copia	5.4	6 (3)	19.3	2 (0)	0.4
Copia_7	LTR-retrotransposon/Copia	5.3	5 (2)	11.1	3 (0)	0.6
Copia_8	LTR-retrotransposon/Copia	5.2	4 (2)	11.0	7 (1)	7.6
Copia_9	LTR-retrotransposon/Copia	5.3	5 (1)	8.8	3 (0)	2.8
Copia_10	LTR-retrotransposon/Copia	5.5	2 (1)	5.8	5 (0)	9.5
Copia_11	LTR-retrotransposon/Copia	5.4	3 (1)	5.7	9 (1)	8.2
Copia_12	LTR-retrotransposon/Copia	1.4	17 (1)	4.3	14 (0)	2.4
Copia_13	LTR-retrotransposon/Copia	5.3	3 (0)	4.0	4 (1)	5.6
Copia_14	LTR-retrotransposon/Copia	5.4	2 (0)	2.9	3 (1)	7.9
Copia_15	LTR-retrotransposon/Copia	5.3	2 (0)	2.0	5 (1)	8.0
Copia_16	LTR-retrotransposon/Copia	5.2	3 (0)	0.3	5 (1)	6.0
Copia_17	LTR-retrotransposon/Copia	1.0	0 (0)	0.0	3 (1)	1.5
DIRS_1	LTR-retrotransposon/DIRS	4.8	22 (2)	19.1	14 (0)	6.6
DIRS_2	LTR-retrotransposon/DIRS	3.7	7 (3)	14.1	11 (4)	21.5
DIRS_3	LTR-retrotransposon/DIRS	1.3	6 (1)	3.7	13 (5)	9.2
DIRS_4	LTR-retrotransposon/DIRS	2.0	0 (0)	0.0	1 (1)	2.0
Gypsy_1	LTR-retrotransposon/Gypsy	6.7	56 (31)	252.4	16 (0)	17.0
Gypsy_2	LTR-retrotransposon/Gypsy	6.7	46 (24)	212.5	12 (1)	4.2
Gypsy_3	LTR-retrotransposon/Gypsy	9.4	54 (18)	192.9	64 (0)	59.8
Gypsy_4	LTR-retrotransposon/Gypsy	11.3	26 (7)	109.9	13 (1)	23.2
Gypsy_5	LTR-retrotransposon/Gypsy	7.0	34 (6)	98.2	34 (3)	70.2
Gypsy_6	LTR-retrotransposon/Gypsy	7.2	29 (1)	63.2	40 (0)	40.4
Gypsy_7	LTR-retrotransposon/Gypsy	7.3	39 (7)	59.5	19 (0)	8.8
Gypsy_8	LTR-retrotransposon/Gypsy	12.5	16 (3)	45.0	13 (0)	9.9
Gypsy_9	LTR-retrotransposon/Gypsy	12.1	41 (1)	39.8	49 (0)	18.0
Gypsy_10	LTR-retrotransposon/Gypsy	8.8	29 (1)	33.7	21 (0)	10.4
Gypsy_11	LTR-retrotransposon/Gypsy	6.9	23 (1)	33.4	14 (0)	2.5
Gypsy_12	LTR-retrotransposon/Gypsy	9.3	6 (3)	33.1	3 (0)	0.4
Gypsy_13	LTR-retrotransposon/Gypsy	10.3	14 (3)	32.1	6 (1)	12.1
Gypsy_14	LTR-retrotransposon/Gypsy	9.4	5 (2)	30.8	1 (1)	9.4
Gypsy_15	LTR-retrotransposon/Gypsy	12.9	16 (1)	28.4	14 (1)	29.2
Gypsy_16	LTR-retrotransposon/Gypsy	9.9	8 (1)	25.5	7 (1)	13.0
Gypsy_17	LTR-retrotransposon/Gypsy	3.4	29 (1)	25.2	21 (0)	4.0
Gypsy_18	LTR-retrotransposon/Gypsy	7.7	22 (2)	24.9	11 (0)	6.3
Gypsy_19	LTR-retrotransposon/Gypsy	11.2	3 (2)	22.7	7 (3)	36.2
Gypsy_20	LTR-retrotransposon/Gypsy	9.2	7 (2)	21.8	5 (0)	10.0
Gypsy_21	LTR-retrotransposon/Gypsy	8.9	5 (2)	21.4	4 (0)	22.4
Gypsy_22	LTR-retrotransposon/Gypsy	9.6	4 (2)	20.9	3 (2)	19.3
Gypsy_23	LTR-retrotransposon/Gypsy	9.6	22 (1)	20.2	17 (0)	7.3
Gypsy_24	LTR-retrotransposon/Gypsy	9.5	4 (1)	19.2	3 (2)	19.4
Gypsy_25	LTR-retrotransposon/Gypsy	7.4	31 (1)	17.0	24 (0)	12.8
Gypsy_26	LTR-retrotransposon/Gypsy	4.2	12 (2)	15.7	16 (0)	2.4
Gypsy_27	LTR-retrotransposon/Gypsy	8.0	19 (1)	14.8	20 (1)	12.2
Gypsy_28	LTR-retrotransposon/Gypsy	9.0	4 (1)	14.7	1 (0)	0.3
Gypsy_29	LTR-retrotransposon/Gypsy	10.0	9 (2)	14.6	10 (0)	14.1
Gypsy_30	LTR-retrotransposon/Gypsy	7.8	10 (0)	14.1	13 (1)	19.2
Gypsy_31	LTR-retrotransposon/Gypsy	5.4	27 (0)	11.7	37 (1)	19.3
Gypsy_32	LTR-retrotransposon/Gypsy	11.5	2 (1)	11.6	2 (0)	0.3
Gypsy_33	LTR-retrotransposon/Gypsy	8.3	13 (1)	11.4	7 (1)	10.6
Gypsy_34	LTR-retrotransposon/Gypsy	8.3	7 (1)	11.1	3 (1)	11.0
Gypsy_35	LTR-retrotransposon/Gypsy	9.2	9 (1)	10.3	11 (1)	15.3
Gypsy_36	LTR-retrotransposon/Gypsy	9.6	2 (1)	9.8	1 (0)	0.3
Gypsy_37	LTR-retrotransposon/Gypsy	9.5	2 (1)	9.7	2 (0)	0.2
Gypsy_38	LTR-retrotransposon/Gypsy	7.9	7 (1)	9.6	4 (1)	11.8
Gypsy_39	LTR-retrotransposon/Gypsy	9.1	3 (1)	9.5	2 (0)	0.3
Gypsy_40	LTR-retrotransposon/Gypsy	10.5	14 (1)	9.3	8 (1)	12.2
Gypsy_41	LTR-retrotransposon/Gypsy	4.9	20 (1)	14.1	17 (0)	3.9
Gypsy_42	LTR-retrotransposon/Gypsy	6.1	11 (1)	10.5	13 (0)	1.7
Gypsy_43	LTR-retrotransposon/Gypsy	4.7	14 (1)	6.7	9 (0)	1.1
Gypsy_44	LTR-retrotransposon/Gypsy	1.0	6 (6)	6.3	5 (5)	5.2
Gypsy_45	LTR-retrotransposon/Gypsy	8.4	6 (0)	4.0	14 (1)	11.8
Gypsy_46	LTR-retrotransposon/Gypsy	5.6	5 (0)	1.4	7 (1)	7.1
Gypsy_47	LTR-retrotransposon/Gypsy	9.1	10 (0)	0.8	11 (1)	13.7
Gypsy_48	LTR-retrotransposon/Gypsy	6.2	2 (0)	0.6	1 (1)	6.2
Gypsy_49	LTR-retrotransposon/Gypsy	5.7	4 (0)	0.5	4 (1)	6.3
Gypsy_50	LTR-retrotransposon/Gypsy	8.9	1 (0)	0.3	1 (1)	8.9
Gypsy_51	LTR-retrotransposon/Gypsy	9.7	2 (0)	0.1	3 (1)	10.1
Gypsy_52	LTR-retrotransposon/Gypsy	2.5	2 (1)	0.1	1 (0)	0.0
Gypsy_53	LTR-retrotransposon/Gypsy	2.8	0 (0)	0.0	1 (1)	2.8
LINE_1	Non-LTRretrotransposon/L1	5.4	14 (4)	30.9	17 (4)	39.6
LINE_2	Non-LTRretrotransposon/L1	2.5	23 (2)	13.7	14 (0)	8.2
LINE_3	Non-LTRretrotransposon/L1	3.8	3 (0)	2.1	6 (1)	6.8
HELPO2	DNAtransposon/Helitron	6.4	15 (5)	44.4	20 (2)	24.0
HELPO1	DNAtransposon/Helitron	7.2	14 (6)	44.9	4 (0)	4.2
TIR_1	DNAtransposon/ Tc1-mariner	1.6	10 (3)	7.3	21 (3)	11.4
TOTAL REPEATS			1051 (204)	2119.2	873 (65)	892.7
Genome percentage (known families)				6.20%		2.50%
Genome percentage (unknown repeats)				3.60%		2.30%

* RepeatMasker reconstructed copies. Full-length copies are shown in parenthesis (>90% length over family consensus).

The *P*. *ostreatus* repetitive element landscape was clearly dominated by Class I transposons, which accounted for 93% of the total TE content in PC15 and 89% in PC9. LTR-retrotransposons were the most abundant TE order, and were responsible for the main differences in TE content between PC15 and PC9. In fact, the four largest Gypsy families (Gypsy_1, Gypsy_2, Gypsy_3 and Gypsy_4) accounted for 2.2% of the PC15 genome size, but only 0.3% in the case of PC9. In addition, these families displayed 80 full-length copies in the former, whereas only fragments and two full-length copies were found in the latter (Table 1). A similar situation occurred with the most prominent Copia families (Copia_1 and Copia_2). Despite the important differences found between PC15 and PC9 in the number of full-length copies and the amount of LTR-retrotransposon masked sequences, the total number of detected TE fragments was closer (1,051 in PC15 *vs* 873 in PC9). The same was true with the amount of solo-LTRs (609 in PC15 *vs* 585 in PC9). Non-LTR retrotransposons (L1 elements) were found in similar abundance in PC9 and PC15, although at lower copy numbers than LTR-retrotransposons. The repertoire of Class II elements found in the genomes was dominated by the previously described Helitron families HELPO1 and HELPO2 [37]. In addition, we identified a family of Tc1-mariner transposons (TIR_1) showing putative autonomous elements as well as non-autonomous truncated copies. Autonomous elements of the latter family were present in both genomes, encoding a transposase carrying DDE3 endonuclease (pfam13358) and Tc3 transposase (cl09264) domains. Additionally, TIR_1 elements show terminal inverted repeats of 214 nt and generate a 2bp target site duplication (TA) upon insertion. Full TE annotations in PC15 and PC9 assemblies are deposited in the Supplementary Information (S1 and S2 Datasets, respectively).

### Estimation of PC9 TE content from 454 sequencing reads

Our screening of TE sequences in *P*. *ostreatus* genome assemblies uncovered that some of the most important LTR-retrotransposon families of PC15 were under-represented in PC9 (Table 1). We hypothesized that our estimation of TE content in PC9 could be underestimated in comparison to PC15 due to its lower assembling quality. In order to know whether this TE families were present in the genome but couldn’t be properly assembled, we analyzed the TE content of PC9 clean 454 sequencing reads (read length of 80 to 626 nt, median length of 364 nt). Datasets of 1.58x and 1.76x genome coverages were randomly sampled from two sequenced libraries, and repeat-masked using our curated TE library to provide an unbiased estimation of TE content. The analysis yielded an average TE content of 4.98%, being the amount of sequence masked by each TE family highly correlated between the two datasets (R^2^ = 0.98, S3 Dataset). In addition, the results showed that Gypsy_1, Gypsy_2 and Gypsy_3 LTR-retrotransposon families were the most abundant in PC9 genome, similarly to that found in the fully assembled PC15 strain.

### TE distribution across the *P*. *ostreatu*s genome

**Fig 2:**
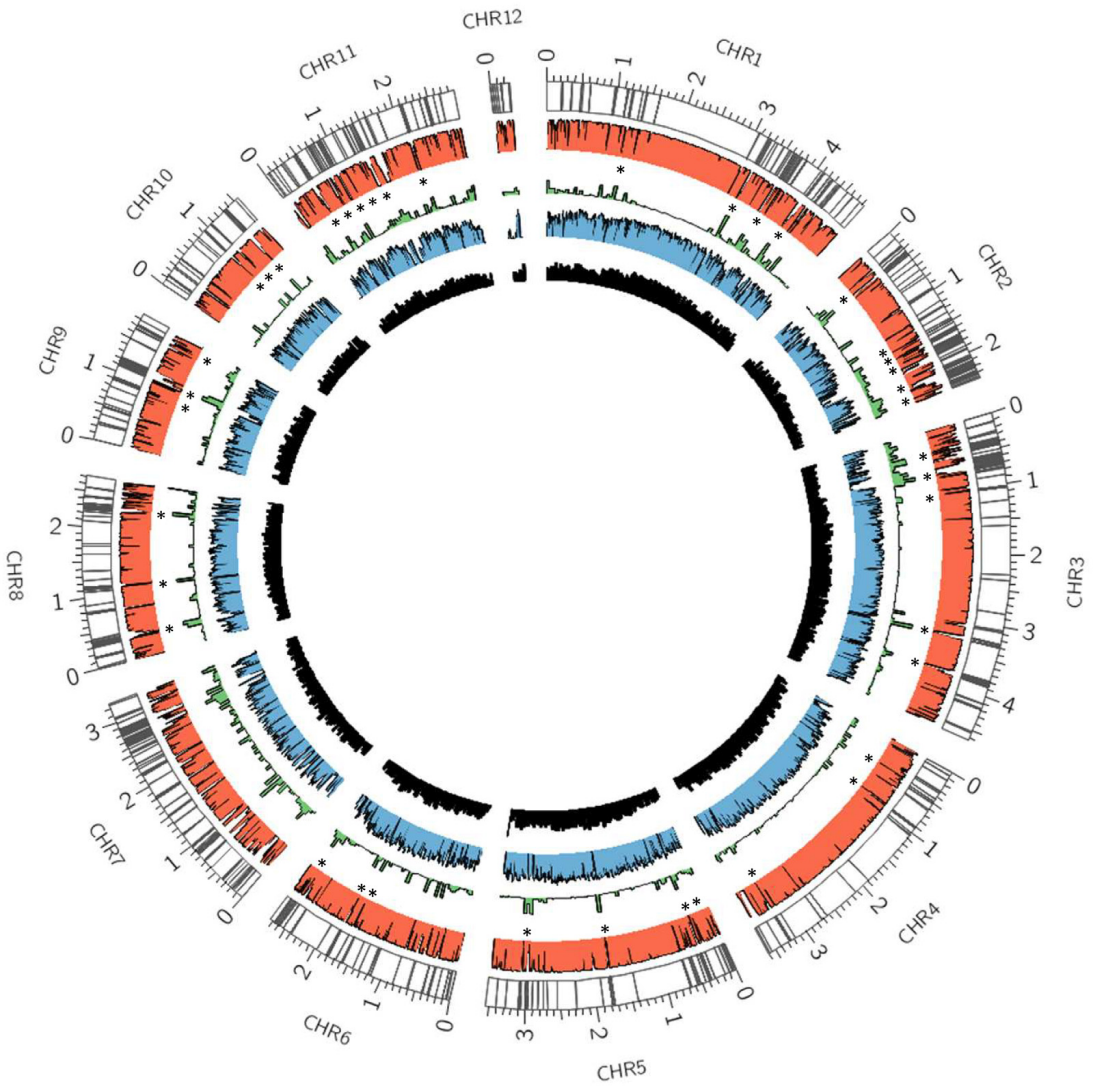
Distribution of transposable elements in the *P*. *ostreatus* genome and transcriptome context. Each band represents the presence of a transposable element. The PC15 –PC9 genome alignment is shown in red, as a histogram of similarity. Coverage of all repeats (including known and unknown families), transcriptome, and gene densities are shown in green, blue and black histograms. Asterisks indicate regions significantly enriched in TEs (p < 0.05).

The density of TEs in *P*. *ostreatus* was highly variable among the twelve chromosomes and regionally within each chromosome (Fig 2). TEs were not randomly distributed over the genome (Mann-Whitney-Wilcoxon p = 2.2e-16), and overlapped frequently with annotated genes (502 in PC15 and 339 in PC9, hereafter referred as “TE-associated genes”). The results of a hypergeometric test performed on the fully assembled PC15 strain revealed that 58% of the TEs were arranged in retrotransposon-rich clusters showing poor sequence conservation between the two genomes. A total of 2,108 genes out of 12,330 were present in these repeat-rich regions. Of these genes, 70 were annotated as lignocellulose-degrading enzymes such CAZymes, manganese and versatile peroxidases, although their presence in TE clusters was not over-represented in comparison to the whole genome (Fisher p value = 0.52). At an inter-specific level, the impact of TE insertions was even more striking, as the conservation of these transposon-enriched regions drops dramatically compared with other basidiomycetes (S1 Fig).

**Fig 3:**
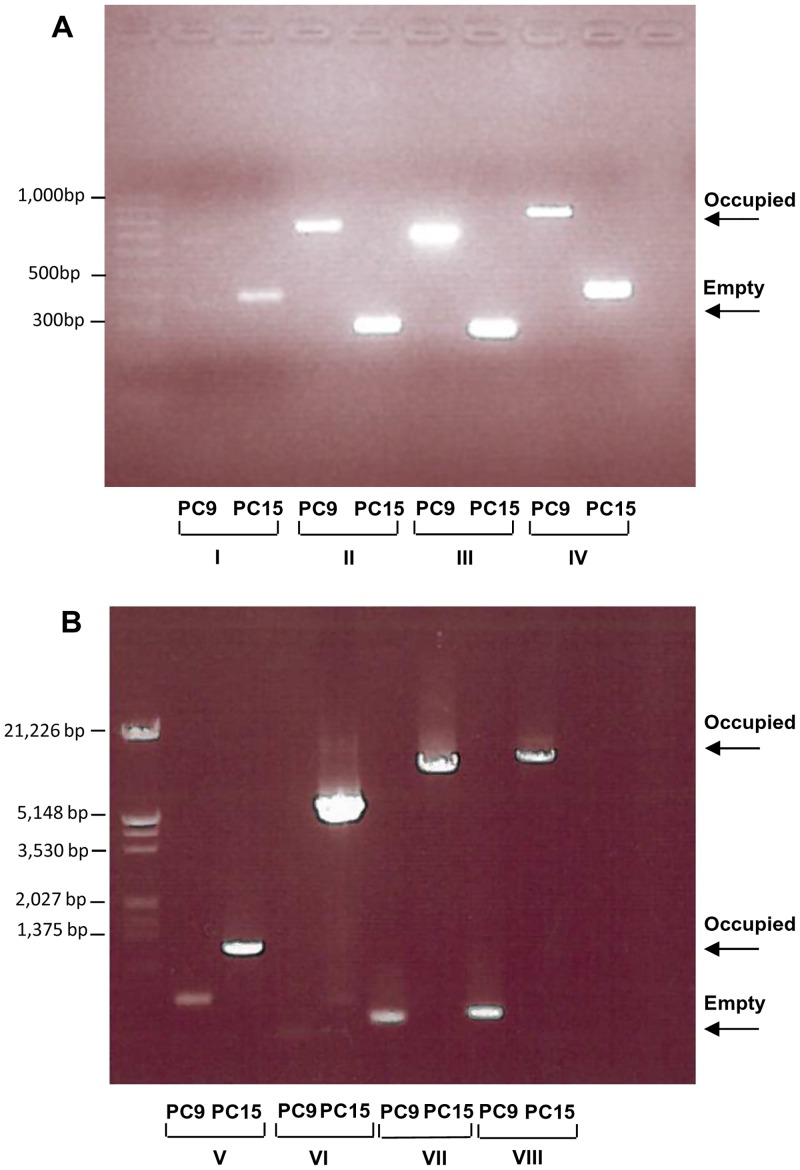
Molecular validation of polymorphic insertions in PC15 and PC9 strains. Primers I to VIII were designed to flank heterozygous TE insertions (present only in one of the two genomes for a given locus) and were used to amplify the target loci in both strains (S1 Text). Panel (A) shows TE insertions in PC9 strain, and panel (B) shows TE insertions in PC15.

A whole genome alignment between PC15 and PC9 was performed to detect *in silico* polymorphic TE insertions. The alignment of every TE locus was extracted and parsed to detect the allelic state (genotype) based on the alignability of such regions. We used the same pipeline to analyze the allelic state of 11,630 protein-coding genes. While only 7.7% of the protein coding genes were heterozygous alleles, up to 50% of TE insertions were polymorphic. Bioinformatics predictions were validated by PCR in a subset of eight polymorphic insertions (Fig 3).

### Dynamics of LTR-retrotransposon amplification in *P*. *ostreatus*

**Fig 4:**
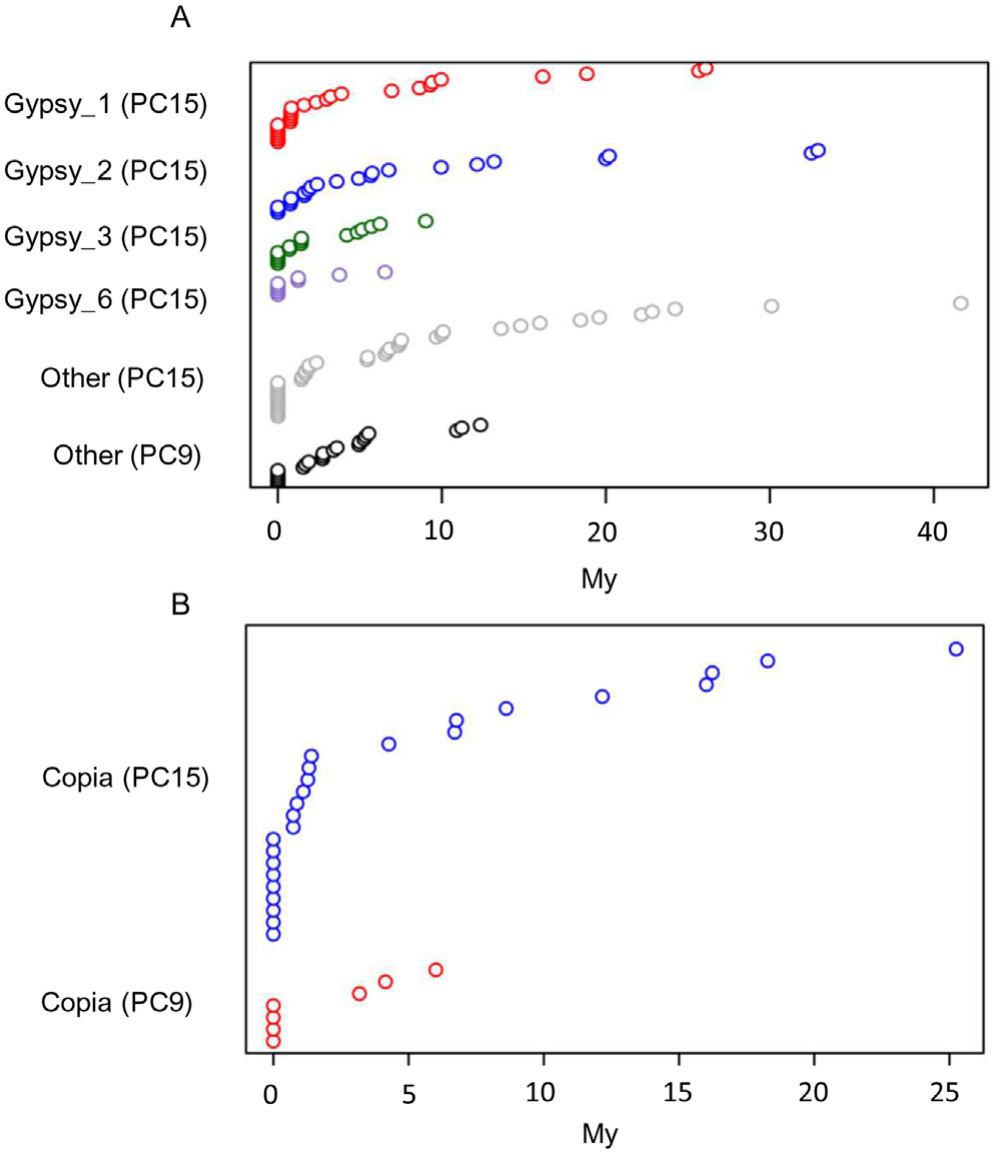
LTR-retrotransposon insertion age in *P*. *ostreatus*. Estimated insertion dates of Gypsy (A) and Copia (B) elements. Each circle represents one element. Families with more than 5 intact copies have their own category in the Y axes. “Other” represents LTR-retrotransposons belonging to smaller families.

The insertion ages of all intact LTR-retrotransposons (carrying both Long Terminal Repeats, n = 189) were estimated based on the nucleotide divergence of LTRs using the approach described in [38] and the fungal substitution rate of 1.05 × 10^−9^ nucleotides per site per year [39,40]. Our results showed that 33% of the LTR-retrotransposon insertions occurred during a recent amplification burst (0 My), and up to 64% were amplified during the last 5 My (Fig 4). The oldest PC15 LTR-retrotransposon insertion clocked 41 My ago, while the oldest element in PC9 clocked 12 My ago. The phylogenetic reconstruction of the LTR-retrotransposon families revealed that some of the most prominent and recently amplified Gypsy families (Gypsy_1, Gypsy_2, Gypsy_5 and Gypsy_6) were phylogenetically close (S2 Fig).

### Transcriptional activity of *P*. *ostreatus* TEs

**Fig 5:**
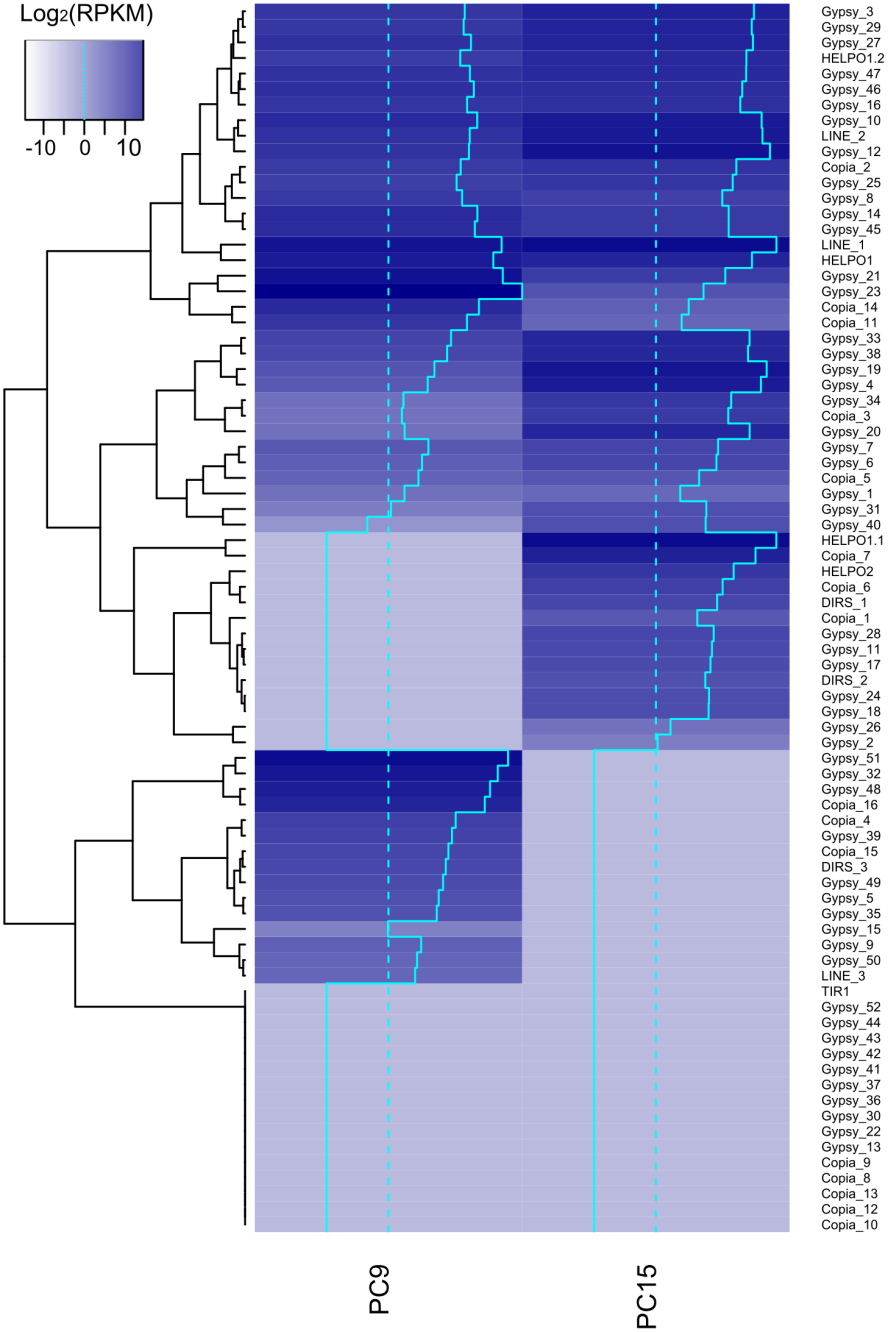
Expression of TE families in *P*. *ostreatus* PC15 and PC9. Heatmap combined with hierarchical clustering showing the transcription of each TE family in LOG_2_(RPKM) normalized per copy number. The blue plain line in the heatmap represents the expression value of each family in the x-axis, and the blue dashed line represents a value of 0 in the x-axis.

We obtained the average expression of every TE family normalized per family size using RNA-seq (Fig 5). Among the main TE groups, LINE was the most abundantly expressed in both strains, followed by Helitrons (especially the HELPO1 family) in PC15 and Gypsy retrotransposons in PC9. At the family level, 60% were expressed in PC15 and 59% in PC9, while at the copy level only 14% and 17% showed transcription, respectively. In addition, 16 out of the 80 families were transcriptionally silent in both strains. Notably, the three strain-specific families in *P*. *ostreatus* (Copia_17, DIRS_4 and Gypsy_53, present only in PC9) were transcriptionally active.

### Impact of TEs on the *P*.*ostreatus* functional genome

**Fig 6:**
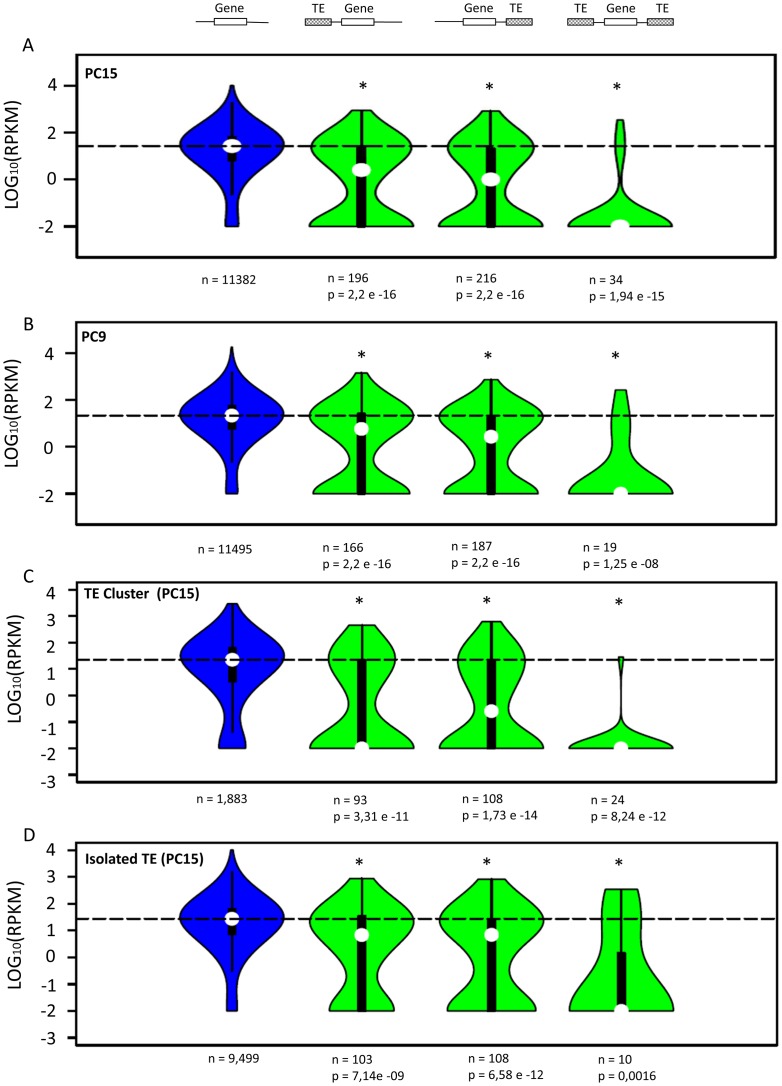
Impact of transposable elements on the expression of neighboring genes in *P*. *ostreatus*. Green violin plots show the expression of PC15 (A) and PC9 (B) genes carrying a TE insertion in the three studied scenarios. Controls in A and B (blue) show the expression of all non-TE genes that are not represented in the other three scenarios. Chart C shows the expression of PC15 genes inside TE clusters. Control (blue) shows the expression of all non-TE genes localized inside TE clusters that are not represented in the other three scenarios. Chart D shows the expression of genes localized outside TE clusters. Control (blue) shows the expression of all non-TE genes localized outside TE clusters that are not represented in the other three scenarios. For every chart, the dotted line shows the median of the control group. White circles inside violin plots represent the median of each distribution. An asterisk indicates that the gene expression distribution of the test group and the control is different (p < 0.05, Mann-Whitney-Wilcoxon test). The number of genes belonging to each distribution is shown under the plot (n).

To investigate the impact of TEs on the functional genome of *P*. *ostreatus*, we explored the effect of TEs on the expression of the surrounding genes. The closest TE insertion to each gene was identified in the three following scenarios (TE-associated genes were excluded from the analysis): i) a TE was present in a 1kb window upstream of the gene start codon, ii) a TE was present in a 1 kb window downstream of the gene end, and iii) a TE was present in both upstream and downstream regions in a window of 1 kb (gene “captured” between two TEs). This window size was selected based on the small intergenic distance of *P*. *ostreatus* (1.14 Kb). When we analyzed the gene expression distribution in every scenario, significant differences were uncovered between controls and genes under TE influence (Fig 6A and 6B). In particular, a strong repression was found for genes captured between two TEs (scenario III), while a discontinuous repression was found when the TE was present upstream or downstream of the gene body (scenarios I and II). In the latter case, distribution shapes indicate that approximately half of the genes were repressed and the other half remained unaltered.

To investigate whether this silencing effect could be influenced by the TE distribution along the chromosomes, we split the analysis of the PC15 strain in two additional scenarios: i) the gene under TE influence was located inside a significant TE cluster (Fig 6C) and ii) the gene under TE influence was located outside a significant TE cluster (isolated TE) (Fig 6D). The results showed that the impact of TEs on gene expression was more intense when insertions occurred inside TE clusters. Additionally, significant differences were found between the distribution of gene expression of genes inside clusters that were not under the influence of TEs (control plot, Fig 6C) and that of the genes in the same condition but outside TE clusters (control plot, Fig 6D, p = 1.22e-8).

To corroborate the hypothesis of TE-mediated gene repression we studied the transcription of orthologous genes displaying polymorphic insertions (always in a window size of 1 Kb), where a TE was present in PC15 and absent in PC9 and *vice versa*. Tables 2 and 3 show 21 genes that were inactive under TE influence and active in the orthologous, TE-free allele. Gypsy LTR-retrotransposons were the main TEs involved in the repression with only two exceptions, which involved the Copia_5 (LTR-retrotransposon) and HELPO1 (Helitron) families. The inactivated genes displayed a broad range of functions. Additional orthologous pairs showing strong repression in the allele under TE influence (5 fold) are shown in S2 Table.

**Table 2:** Expression of orthologous genes displaying TE insertion in PC15. The first two columns are the protein IDs of the JGI *P*. *ostreatus* genome database.

PC9 (no TE)	PC15 (TE)	PC9 RPKM	PC15 RPKM	TE family	Interpro description
101709	1048159	73.6	0	Gypsy_2	Unknown
99511	171575	34.6	0	Gypsy_3	Peptidase M
87521	1085356	9.9	0	Helpo1	Unknown
87521	160117	9.9	0	Gypsy_7	Unknown
63834	1109156	2	0	Gypsy_47	Unknown
67552	1033100	1.6	0	Gypsy_1	Unknown
108646	1103939	1.5	0	Gypsy_3	Unknown

**Table 3:** Expression of orthologous genes displaying TE insertion in PC9. The first two columns are the protein IDs of the JGI *P*. *ostreatus* genome database.

PC9 (TE)	PC15 (no TE)	PC9 RPKM	PC15 RPKM	TE Family	Interpro description
131667	1102590	0	31.61	Gypsy_3	Unknown
95320	1077306	0	26.88	Helpo1	NAD-dependent epimerase/dehydratase
66978	159492	0	26.77	Gypsy_3	RNA polymerase II, large subunit
68190	33483	0	19.06	Gypsy_31	Unknown
131853	49007	0	12.93	Gypsy_18	Serine/threonine protein kinase
132116	1110152	0	10.36	Gypsy_17	Phospholipase A2
108952	1081099	0	9.72	Gypsy_26	Protein kinase
131565	166826	0	9.72	Gypsy_9	F-box
68399	165925	0	9.42	Gypsy_17	ATP-dependent RNA helicase
91452	160772	0	8.02	Copia_5	Unknown
64875	1109777	0	7.26	Gypsy_6	Cyclin-like
66851	160925	0	2.83	Gypsy_31	Unknown
125628	1102342	0	2.76	Gypsy_41	alpha/beta-Hydrolases
102080	159538	0	1.48	Gypsy_40	Unknown

### Differential expansions of transposable elements in fungi

**Fig 7:**
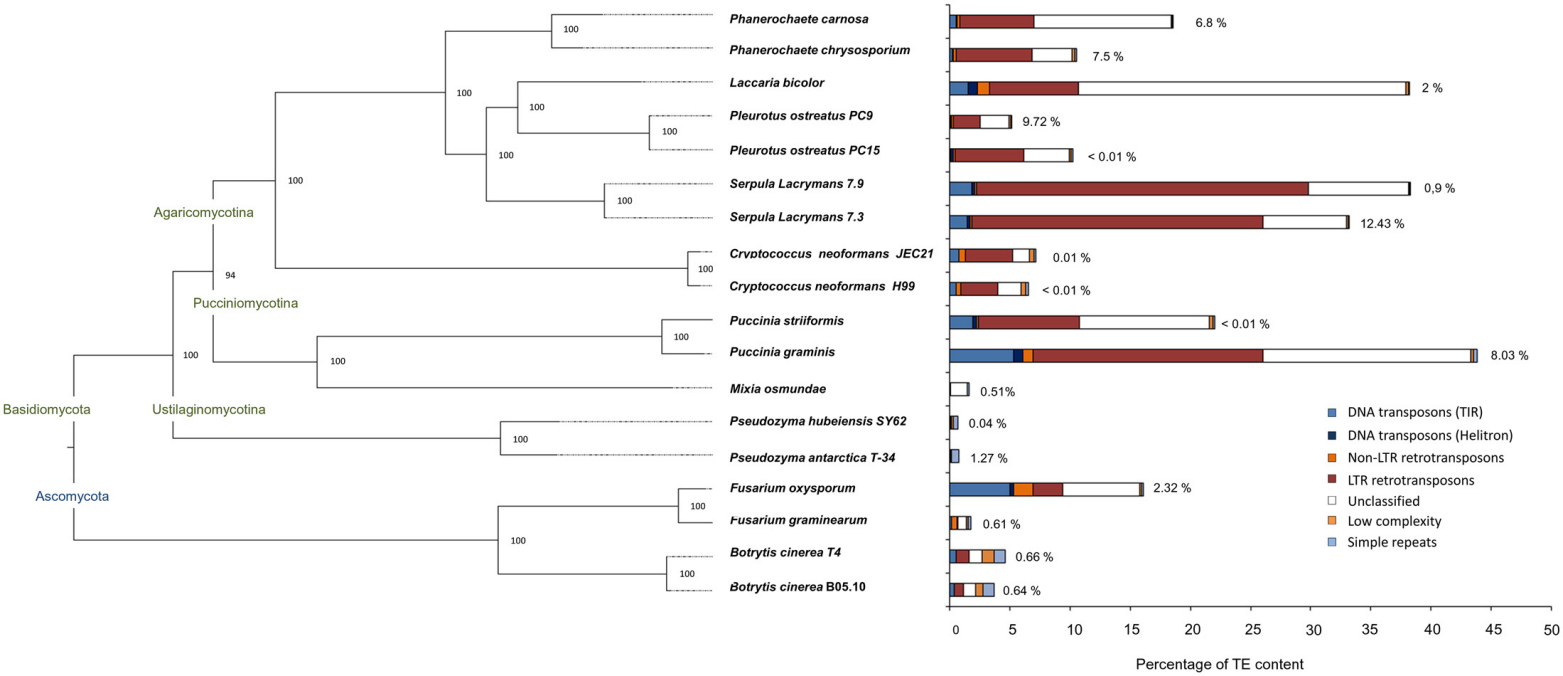
Phylogeny and repeat content of eighteen fungal species. Maximum-likelihood phylogeny inferred with RAxML based on 551 genes and 100 bootstraps. Percentages of assembly gaps are shown near to each bar. Dashed lines are used to align each branch to the tip.

Our pipeline for the identification, classification and annotation of transposable elements was performed in eighteen Ascomycetes and Basidiomycetes genomes (Fig 7). The results demonstrated great variability in TE content at the phylum, genus and species levels (Fig 7, S3 Table). Elements belonging to 20 different TE superfamilies (11 of Class I and 9 of Class II) were identified and classified into the main groups shown in Fig 7. The genome percentage occupied by these TE families showed a positive correlation with genome size (R^2^ = 0.38). Within the genera analyzed, *Serpula* showed a surprisingly high TE content in proportion to its genome size, especially due to LTR-retrotransposon expansions in the Gypsy and Copia superfamilies. In fact, when excluding the two *Serpula* genomes from the analysis, the correlation between TE content and genome size in the remaining species was much higher (R^2^ = 0.71). The Ascomycete species analyzed had a ratio of Class I / Class II elements ranging from 0.78 to 4.23 and a low content of repetitive sequences, with the exception of the plant pathogen *F*. *oxysporum*. Interestingly, this species showed a 15-fold enrichment of transposable elements compared with *F*. *graminearum* as a result of important expansions of Class II elements (Tc1-mariner and hAT families). The variability in the TE content in the analyzed Basidiomycetes ranged from species practically free of TE repeats, such as in the *Pseudozyma* genera (0.02% of the genome), to species with almost one third of their genome masked by the TE library, such as *Serpula lacrymans* or *Puccinia graminis*. TE expansions seemed to be constrained in basidiomycete yeasts such *Pseudozyma* or *Mixia* compared to the rest of the basidiomycetes analyzed. LTR-retrotransposons in the Gypsy and Copia superfamilies families were the main elements responsible for differences in TE content, with the Class I / Class II ratio much higher in basidiomycetes than in ascomycetes (9.3 in average). In fact, these two superfamilies were detected in all species analyzed in this study. When we studied the differential TE amplifications at the genus/species level, we found six pairs that displayed similar content (*Botrytis*, *Cryptococcus*, *Phanerochaete*, *Serpula*, *Pleurotus* and *Pseudozyma*) and two pairs (*Fusarium* and *Puccinia*) that showed important differences between counterparts.

### Impact of transposable elements on neighboring gene expression in other fungal models

**Fig 8:**
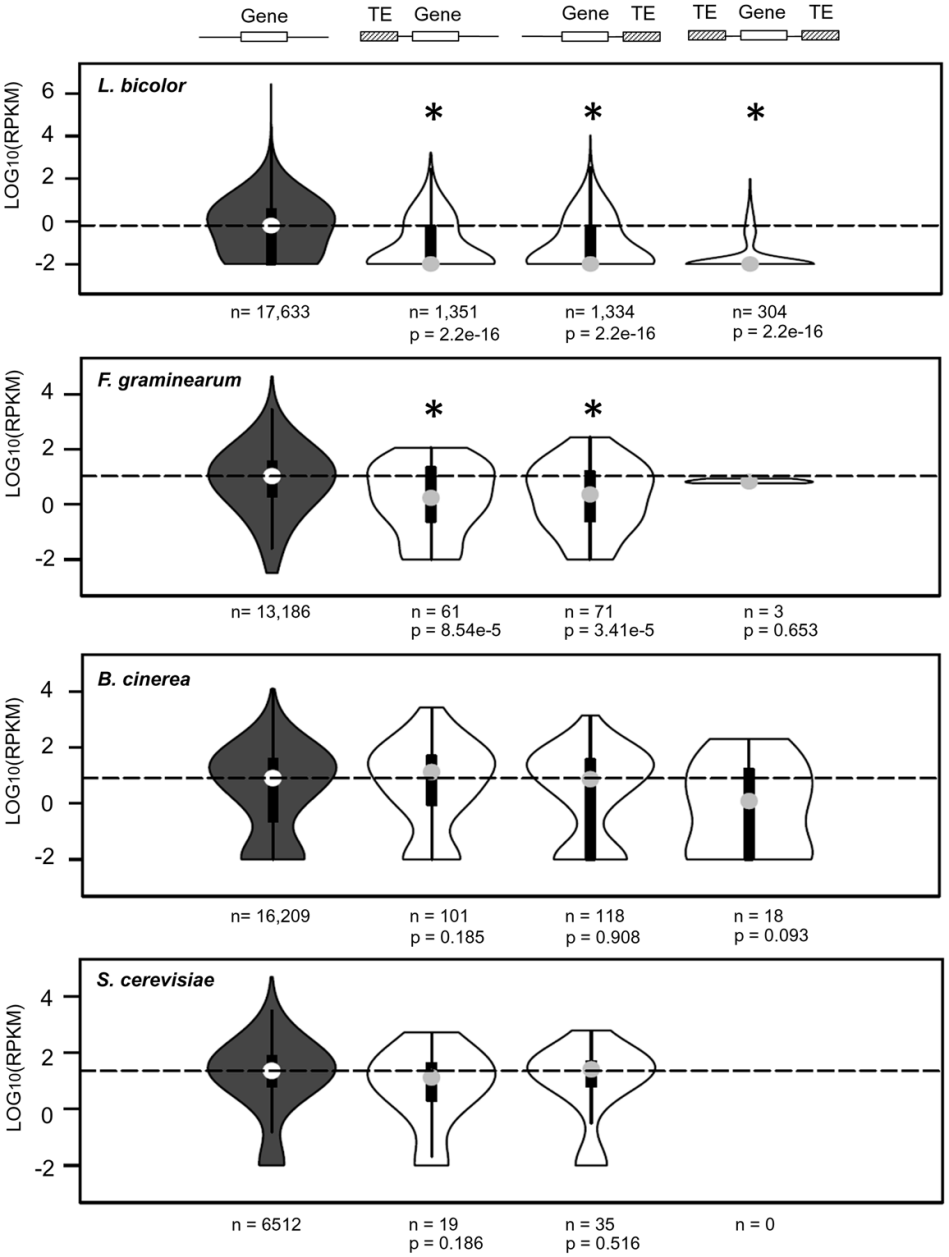
Impact of TE insertions on the expression of the closest gene in four fungal models. *S*. *cerevisiae* TE annotation was obtained from the SGD database (http://www.yeastgenome.org/). An asterisk indicates that the gene expression distribution of the test group (white) and the control (grey) is different (p < 0.05, Mann-Whitney-Wilcoxon test). The number of genes belonging to each distribution is shown under the plot (n).

The effect of TE insertions in nearby genes was analyzed in four additional fungal models: *Laccaria bicolor*, *Fusarium graminearum*, *Botrytis cinerea* B05.10 and *Saccharomyces cerevisiae* S288C. These species were chosen based on the public availability of genomic (full genome sequence) and transcriptomic (RNA-seq) data. In addition, *L*. *bicolor* and *S*. *cerevisiae* were chosen based on their opposite methylation patterns (evidence of methylation *vs* absence of methylation, respectively [11]). The analysis uncovered two clear profiles. First, *L*. *bicolor* and *F*. *graminearum* showed a pattern of TE-mediated repression similar to *P*. *ostreatus*, in which an important number of genes carrying TE insertions within a 1 kb upstream/downstream window were repressed (Fig 8). Second, *B*. *cinerea* and *S*. *cerevisiae* genes under TE influence did not show any alteration in expression, with distributions identical to the control (p > 0.05, Fig 8)

### Horizontal transfer of Tc1-mariner transposons in eukaryotes

**Fig 9:**
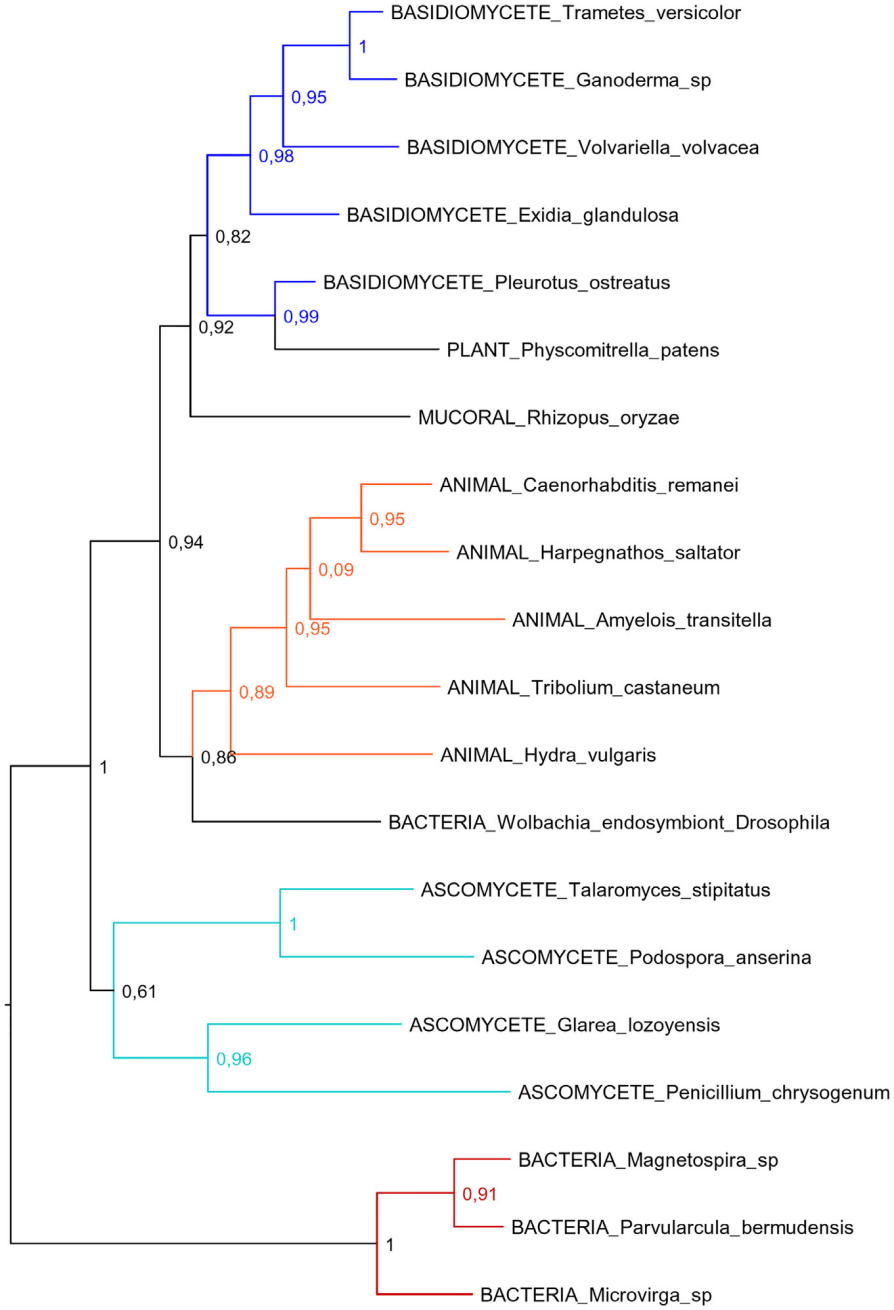
Phylogenetic reconstruction of TIR_1-like Tc1-mariner transposases. Basidiomycete, ascomycete, animal, and bacterial Tc1-mariner transposases are shown in dark blue, light blue, orange and red, respectively. SH indices are included indicating branch support.

During the process of TE classification using BLASTX against Repbase peptide database we noticed high similarity between the *P*. *ostreatus* TIR_1 family and the previously described Mariner2_PPa [41] (71% nucleotide identity over 71% of the sequence), a Tc1-mariner element identified in the moss *Physcomitrella patens*. According to the nucleotide divergence estimated by K2P distance and the fungal nucleotide substitution rate, TIR_1 and Mariner2_PPA diverged 517 My ago, despite mosses and fungi diverged about 1,600 My ago [42]. To investigate if horizontal transfers could have played a role in the distribution of fungal and other eukaryotic Tc1-mariners, we reconstructed the phylogeny of their encoded transposases (Fig 9). Our dataset included fungal, animal, plant and bacterial Tc1-mariner transposases, which were obtained based on best BLAST hits against NCBI and JGI reference proteins databases. The topology of the gene tree shows clear incompatibilities with the phylogenetic relationships of the species analyzed, which might be explained by horizontal transfers of Tc1-mariners. Specifically, basidiomycete and animal transposases were placed in a single clade with very high support, separated from ascomycete transposases. Other phylogenetic incongruences were the presence of the moss *Physcomitrella patens* and the mucoral *Rhizopus oryzae* in the basidiomycete clade, as well as the endosymbiont bacteria *Wolbachia* present in the animal clade.

## Discussion

### TE detection, classification and annotation in *P*. *ostreatus*

Fungal TE content is highly diverse, even within species that are phylogenetically close [28]. However, studies analyzing the intra-specific variability in TE content have been infrequent. According to our results, transposable elements accounted for a small to moderate amount of the genome size in the two *P*. *ostreatus* strains analyzed (6.2% in PC15 and 2.5–4.9% in PC9). Although the number of TEs detected varies according to the pipeline used, the TE content in *P*. *ostreatus* fell within the range reported for most fungal genomes (from 0 to 25%) [15,28,43,44,45], with the exception of some plant pathogens and ectomycorrhizal species that have undergone massive TE amplifications [32,44]. Despite all TE groups are generally more abundant in PC15 than in PC9, major differences between the strains were observed in LTR-retrotransposons. Most of the LTR-retrotransposon families under-represented in PC9 were actually present in the genome, but could not be assembled into the main scaffolds due to its length and repetitive nature. Assembling transposable elements is technically challenging because identical TE copies require sequencing reads exceeding the TE length to be resolved [46]. This is especially relevant in *P*. *ostreatus*, as we show that most of its LTR-retrotransposons underwent a recent amplification burst, thus sharing high nucleotide similarity. The presence of TE sequences in the unassembled reads is common in plants and animals [47,48]. In fungi, a recent study performed on several *Amanita* species identified many TEs that could not be found in the assembled regions, especially Gypsy elements [32]. In addition to the difficulty in assembling TE repeats, their structural complexity, which is caused by internal rearrangements, mutations, nested elements and DNA fragment acquisition events, complicated their identification using generic annotation tools. Our multi-way approach used for TE detection greatly improved the discovery of repeats, as revealed by the number of detected families in our combined TE library (Fig 1A). Using this approach was of particular importance for TE detection in PC9, because families that could not be detected by *de novo* searches in the assembly due to its high gap content could be found in PC15 and thus were present in the TE library.

### Transposable element landscape in *P*. *ostreatus*

*P*. *ostreatus* repeat content is enriched in Class I transposons, especially in the Gypsy and Copia superfamilies. LTR-retrotransposons are divided into five superfamilies, but these two are the most abundant in the fungal kingdom [28,49]. The replicative transposition mechanism of autonomous LTR-retrotransposons makes them efficient genome colonizers because the copy number increases with every transposition event. Autonomous LTR-retrotransposons contain *gag* and *pol* genes flanked by long terminal repeats, and they differ from retroviruses in that they do not have infection capacity [50]. The difference between the Gypsy and Copia superfamilies lies in the order of the internal protease, integrase, reverse transcriptase and RNAse H domains present in the *pol* gene. We also found retrotransposons of the DIRS superfamily, which contains a *gag*, *pol* and tyrosine recombinase ORFs flanked by terminal repeats. This group of TEs is less abundant than other retrotransposons, and it exhibited patchy distribution in the fungal phylogeny [51].

One necessary condition for an active TE family is the presence in the genome of autonomous elements encoding the structural features and protein domains necessary for their own transposition. In this sense, the Gypsy architecture seems to be the most successful, as shown by the number of families and number of full-length copies per family. A second condition for TE transposition is that autonomous elements must be transcribed. We showed that although most genomic regions containing TEs are silenced, about 60% of the TE families showed at least one transcriptionally active copy. Interestingly, Class I transposons show high transcriptional levels, which are essential because they are propagated through RNA intermediates that can be translated into proteins necessary for replication or can act as replication templates. In parallel to the successful amplification of LTR-retrotransposons in *P*. *ostreatus*, the presence of solo-LTRs suggests the occurrence of homologous recombination between LTRs leading to retrotransposons elimination. Class II DNA transposons are less abundant than Class I RNA elements and are represented by the Helitron and Tc1-mariner superfamilies. In a previous work, we reported the presence and structure of the two Helitron families in *P*. *ostreatus* [37]. Helitrons were discovered by bioinformatics approaches in *Arabidopsis thaliana* and *Caenorhabditis elegans* more than a decade ago [7]. Nevertheless, the experimental demonstration of their transposition was not described until very recently [52]. Their rolling-circle transposition mechanism and their ability to capture and amplify gene fragments make them interesting subjects of study. Helitrons are present in all eukaryotic kingdoms [53], although they show patchy distribution in some phylogenetic clades, such as mammals. In plants, they play an important role in genome evolution, introducing functional diversity by creating new genes and isoforms [54]. In this study, we showed that Helitrons are the most abundant DNA transposons in the *P*. *ostreatus* genome and are the second superfamily in transcriptional activity. Our results add a piece of evidence to the fact that this superfamily is actively populating the *P*. *ostreatus* genome. Interestingly, within the 19 described superfamilies of cut and paste DNA transposons, only Tc1-mariner is present in *P*. *ostreatus*. According to our results, this superfamily would be the most efficient fungal cut and paste transposon, as it is the most represented in the species analyzed. Nevertheless, most of the copies present in *P*. *ostreatus* are truncated, and the putative autonomous elements encoding transposases are not expressed in the condition tested. Our phylogenetic reconstruction of TIR_1-like Tc1-mariner transposases shows important discordances with organismal phylogenies, suggesting that horizontal transfer has shaped the distribution of these Class II transposons within the eukaryotic kingdom. Specifically, the presence of animal, plant, bacterial, mucoral and basidiomycete transposases in a monophyletic group separated from ascomycetes supports the hypothesis that multiple horizontal transfers occurred after the divergence of basidiomycetes and ascomycetes, event that took place about 1200 My ago [42]. It is known that transposable elements are horizontally transferred in eukaryotes at a higher frequency than regular genes [55], and this ability allows them to persist in the course of evolution escaping from vertical extinction [56]. Our data suggests that horizontal gene transfer has played an important role in the dynamics of eukaryotic Tc1-mariners. Nevertheless, the diversity of TE copies, their repetitive nature and the limitations of the taxonomic sampling make difficult to reconstruct the full evolutionary history of TIR_1-like Tc1-mariner transposases.

### Transposable elements in fungi: Burden or opportunity?

Most fungal species have streamlined, compact genomes. Owing to international efforts and advances in genome sequencing over the last decade, there is genomic information for nearly 500 fungal species covering most of the fungal phylogenetic diversity, with more being produced (http://1000.fungalgenomes.org). The assembled genome sizes in fungi range from about 2 to 190 Mb, while flow cytometry estimations have uncovered genome sizes of up to 893 Mb in the Pucciniomycotina subphylum [57] (*Gymnosporangium confusum*). The available data demonstrate the impressive variability in fungal genome size, and our results suggest that an important part of this variability could be explained by differential expansions of TEs that seem to be related to the fungal lifestyle. Our results confirm that obligate biotrophs such *P*. *graminis* and *P*. *striiformis* are highly enriched in TEs [45]. By contrast, the (not obligate) biotroph *M*. *osmundae* is practically free of TEs, similarly to other basidiomycete yeasts such the *P*. *hubeiensis* and *P*. *antarctica*. Previous studies have shown that TE-driven expansions have played important roles in the genomes of filamentous plant pathogens [58]. An example of the impact of TEs in host adaptation and pathogen aggressiveness is the *Leptosphaeria genus* [59]. According to [58], faster adaptation occurs because genes encoding proteins for host interactions are frequently polymorphic and reside within repeat-rich regions of the genome. Due to the presence of *P*. *ostreatus* lignin degrading enzymes within TE clusters, is tempting to hypothesize that TEs could play an important role in the evolution of wood decayers.

### Impact of TEs on genome architecture and functionality

Transposable elements are undoubtedly an important source of genetic variation in fungi. As previously found in other fungal species [43], *P*. *ostreatus* TEs are preferentially arranged in non-homologous genomic regions that display low conservation at both the intraspecific and interspecific levels. These genomic blocks are hotspots for LTR-retrotransposon accumulation, which could target these regions due to specific chromatin structures adopted by pre-existing elements [60].

The compatible monokaryotic strains PC9 and PC15 can mate to form a dikaryon, the nuclei of which coexist in the same cell [35]. Thus, the unpaired long blocks of repetitive DNA are unlikely to undergo crossover and are likely inherited as supergenes after meiosis. We show that the transcription of these TE-rich regions tended to be strongly repressed (Figs 2 and 6) and we hypothesize that genes with essential functions might eventually be captured and silenced during the formation of these TE clusters, leading to a looseness of fit by the monokaryotic genotypes carrying these genomic regions. Selection against these TE blocks would lead to the loss of these alleles in the course of evolution. On the other hand, the higher plasticity of these repeat regions might create novel opportunities for diversification and adaptation. In addition to the permanent genomic modifications that TEs can promote, we showed that both isolated and clustered TE insertions modulate the expression of surrounding genes. In addition to the disruption-mediated changes originated by TE insertions into promoter regions, there are additional mechanisms by which TEs can alter the expression of surrounding genes. TEs often carry *cis*-regulatory elements that can be spread over the genome [26]. Similarly, LTR-retrotransposons and solo-LTRs contain promoters that can activate the expression of dormant genes [60]. Additionally, transcripts from full-length TEs can read through into a neighbor gene, producing spurious transcripts that can be subjected to transcriptional and post-transcriptional control [61]. Finally, TEs can be targeted for heterochromatin formation, thus potentially silencing the transcription of the adjacent gene [26]. Several studies have shown that *Arabidopsis* genes close to TEs had lower expression than the average genome-wide expression [62, 63]. Similarly, a recent study showed that the insertion of SINE retrotransposons close to human and mouse gene promoters led to transcriptional silencing mediated by the acquisition of DNA methylation [64]. The few studies available on the subject in fungi indicate that methylation targets transposon sequences selectively, leading to TE transcriptional silencing [11,17,18]. Although methylation within fungal genes tends to be low, studies in the plant pathogen *Magnaporthe oryzae* showed that genes that were methylated in upstream or downstream regions resulted in lower transcription than un-methylated genes [17]. We hypothesize that the transcriptional repression of genes surrounded by TE insertions could be related to the epigenetic status of the given TE. In fact, the discontinuous repression found in *P*. *ostreatus* genes under TE influence (gene repressed *vs* non-repressed) fits with the putative methylated *vs* non-methylated status of the involved TEs. Although we lack experimental evidence of methylation in PC15 or PC9, the presence in both strains of transcriptionally active homologs of the *Dim-2* DMTase (S3 Fig) responsible for cytosine methylation in fungi [65] suggests that the methylation machinery is active in *P*. *ostreatus*. In addition to *P*. *ostreatus*, we used the same transcriptional analysis pipeline in two species with well-known methylation profiles [11]: *S*. *cerevisiae* (methylation-free) and *L*. *bicolor* (TE regions highly methylated). The expression distribution of *S*. *cerevisiae* genes under TE influence was identical to the control (p < 0.05), while the distribution in *L*. *bicolor* showed a severe bias towards low expressed genes. Additional analyses performed in other species uncovered that the ascomycetes *F*. *graminearum* and *B*. *cinerea* showed different expression patterns for genes under TE influence. Whereas *B*. *cinerea* genes remained unaltered, the expression in *F*. *graminearum* genes was lower than the control. Bisulfite sequencing of *Gibberella zeae* (anamorph: *F*. *graminearum*) showed that this species has low cytosine methylation levels, although it displays related mechanisms of TE silencing, such as RIP and meiotic silencing [66]. Regarding *B*. *cinerea*, the unique reference found on the subject showed that no or very little methylation occurred in this species, according to HpaII/MspI restriction patterns [67]. In summary, we show that transposable element dynamics differentially impact fungal genome-wide transcription patterns, likely as a result of the epigenetic machinery evolved to control TE proliferation.

## Materials and Methods

### Fungal genomes

Eighteen Ascomycetes and Basidiomycetes species were selected in this study as sample sets of closely related species for genomes comparisons. Publicly available genomic assemblies were downloaded from the Joint Genome Institute’s fungal genome portal MycoCosm [68] (http://jgi.doe.gov/fungi), the Broad Institute (https://www.broadinstitute.org/) and FungiDB [69]. The genome sequences of the *P*. *ostreatus* monokaryotic strains PC15 v2.0 [34] and PC9 v1.0, which were obtained by de-dikaryotization of the dikaryotic strain N001 [35], were used as models for building the pipelines described in this paper.

### Identification, classification and annotation of transposable elements (TEs)

*De novo* identification of repetitive sequences in the genome assemblies was performed by running the RECON [70] and RepeatScout [71] programs (integrated into the RepeatModeler pipeline). LTRharvest [36] was used to improve the detection of full length LTR-retrotransposons. LTRharvest results were filtered to avoid false positives as follows: elements were de-duplicated and used as queries for BLASTN searches (cutoff E-value = 10^−15^) against the genome assembly and for BLASTX (cutoff E-value = 10^−5^) against the Repbase peptide database [54]. Only sequences longer than 400 bp with more than five copies or yielding a significant hit to a described LTR-retrotransposon were kept for further analysis. The outputs of the above programs were merged and clustered at 80% similarity using USEARCH [72] to create species-specific (i.e., *P*. *ostreatus* PC15 and PC9) or genus-specific (i.e., *F*. *oxisporum* and *F*. *graminearum*) TE libraries. Each consensus sequences library was classified using BLASTX against the Repbase peptide database, and the final libraries were used as input for RepeatMasker (http://www.repeatmasker.org). Consensus sequences without similarity to any Repbase entry were labeled as ‘unknown’. The RepeatMasker output was parsed using the *One*_*code*_*to*_*find*_*them*_*all* script [73] to reconstruct TE fragments into full-length copies and estimate the fraction of the genome occupied by each TE family.

To identify solo-LTRs, the left terminal repeat of every autonomous copy was extracted, and a BLASTN against each assembly was performed. The flanking sequences of every hit (5,000 bp, cutoff E-value = 10^−15^) were extracted and screened for retrotransposon internal sequences. Solo-LTRs were defined as those hits lacking internal retrotransposon sequences at the flanking sites.

### Analysis of TE distribution in *P*. *ostreatus*

To determine whether TEs were non-randomly distributed, the distribution of inter-TE distances was compared (Mann-Whitney-Wilcoxon text) with that of the inter-element distances of a randomly generated subset of 1,196 elements. In addition, TEs and gene model annotations were merged and used as reference for a hypergeometric test to test for the presence of regions enriched in TEs. The analysis was performed using REEF [74] with a Q-value of 0.05 (FDR 5%), a window width of 100 kb with a shift of 10 kb and a minimum number of 10 features in clusters.

### Whole-genome alignment

The *P*. *ostreatus* PC15 and PC9 genome assemblies were aligned using the Mercator and MAVID pipeline [75], using the fully assembled PC15 genome as a reference. Gene model positions and TE hits of the PC15 strain were used to extract individual alignments and to check the homozygous *vs*. heterozygous nature of the insertions. A locus was considered homozygous if the alignment spanned at least 80% of the whole locus length, and heterozygous when the PC9 allele was absent.

### Estimation of LTR-retrotransposon insertion dates

Long Terminal Repeats of every intact, full-length element were extracted and aligned. Kimura 2-Parameter distance was obtained using a Python script and transformed to My using the approach described in [39] and the fungal substitution rate of 1.05 × 10^−9^ nucleotides per site per year [40].

### Nucleic acid extraction, manipulation and sequencing

Mycelia were harvested, frozen and ground in a sterile mortar in the presence of liquid nitrogen. DNA was extracted using a Fungal DNA Mini Kit (Omega Bio-Tek, Norcross, GA, USA). Sample concentrations were measured using a Qubit 2.0 Fluorometer (Life Technologies, Madrid, Spain), and purity was measured using a NanoDrop 2000 (Thermo-Scientific, Wilmington, DE, USA). PCR reactions were performed according to Sambrook *et al* [76] using primers designed after the TE flanking sequences (S1 Text, Supplementary Information). Total RNA was extracted from 200 mg of deep frozen tissue using Fungal RNA E.Z.N.A Kit (Omega Bio-Tek, Norcross, GA, USA), and its integrity was estimated by denaturing electrophoresis on 1% (w/v) agarose gels. Nucleic acid concentrations were measured using a Nanodrop 2000 (Thermo Scientific, Wilmington, DE, USA), and the purity of the total RNA was estimated by the 260/280 nm absorbance ratio. Messenger RNA was purified using a MicroPoly(A) Purist kit (Ambion, USA). Transcriptome libraries were generated and sequenced by Sistemas Genomicos S.L. (Valencia, Spain) on a SOLiD platform, following the manufacturers’ recommendations (Life Technologies, CA, USA).

### RNA-seq data analysis

*P*. *ostreatus* RNA-seq datasets corresponding to PC15 and PC9 strains (8.4 and 9.7 million reads in PC15 and PC9, respectively) cultured in SMY medium and harvested during the exponential growth phase, were used to analyze the transcription of genes and TEs. The quality of the SOLiD RNA-seq reads was verified using FastQC (http://www.bioinformatics.babraham.ac.uk/projects/fastqc/), and they were mapped to their corresponding PC15 v2.0 or PC9 v1.0 assemblies using TopHat [77], restricting the multihits option to 1. HTseq-count [78] was used to determine the number of reads mapping to every feature. SAMtools [79], BEDTools [80] and custom Python scripts were used to manipulate the data, to calculate RPKMs and to obtain genome coverages. Public RNA-seq data from other species were downloaded from the NCBI SRA database and were analyzed using the same pipeline (accessions SRR1257938 *Saccharomyces cerevisiae* S288C [81], SRR1284049 *Botrytis cinerea* B05.10 [82], SRR1592424 *F*. *graminearum* [83] and SRR1165053 *Laccaria bicolor* [84]).

For analyzing the expression of TE families, reads were mapped to the extracted transposon sequences using Bowtie [85] and allowing multi-mapping. RSEM software was used to calculate TE expression because its algorithm is especially designed to handle multi-mapped reads [86]. Afterwards, the FPKMs of each family were normalized to the number of elements.

### Effect of TE insertions on the expression of downstream genes

Gene and TE annotations were intersected to obtain TE-associated genes (genes overlapping with any TE) and non-TE genes (genes not overlapping with any TE). Afterwards, the closest TE upstream and downstream to each non-TE gene was obtained at a maximum distance of 1 kb. The resulting genes were organized in three groups: i) genes with an upstream TE, ii) genes with a downstream TE and iii) genes with both upstream and downstream TEs. Control groups were obtained by subtracting target genes (three previous scenarios) to all the non-TE genes.

### Phylogenetic analysis of the species used in this study

The predicted proteomes of all species were downloaded from the Mycocosm database (http://genome.jgi.doe.gov/programs/fungi/index.jsf). After all-by-all BLASTP, proteins were clustered with MCL [87] using an inflation value of 2. Clusters containing single copy genes of each genome were retrieved (allowing two missing taxa per cluster) and proteins were aligned with MAFFT [88]. The alignments were concatenated after discarding poorly aligned positions with Gblocks [89]. Maximum-likelihood phylogeny was constructed using RaxML [90] under PROTGAMMAWAGF substitution model and 100 rapid bootstraps.

### Phylogenetic reconstruction of Tc1-Mariner transposases

Using the *P*. *ostreatus* JGI browser we identified the internal transposase gene of a full length element of TIR_1 family. This protein was used as query for BLASTP searches (cutoff = E^-5^) against NCBI RefSeq protein database (independent searches were carried out against animal, plant and bacterial databases). The best five animal, plant and bacterial hits were retrieved when possible (i.e. only one hit was obtained using plant database). The same search was performed in the JGI database to retrieve the best five basidiomycete hits, and the best five non-basidiomycete hits. Proteins were aligned with MUSCLE [91], and the alignments were trimmed using trimAl [92] with the default parameters. An approximate maximum likelihood tree was constructed using FastTree [93] and edited with Figtree (http://tree.bio.ed.ac.uk/software/figtree/). Transposases from *P*. *patens*, *Wolbachia* and *Rhizopus oryzae* were further analyzed to exclude the possibility of being a result of database contamination: Using TBLASTN against NCBI Whole-genome shotgun contigs or JGI genomic scaffolds, we identified their genomic position and verified that they were assembled in long scaffolds and surrounded by other host genes.

### Accession numbers

Raw sequencing data was deposited in GEO database under the accession number GSE81586.

## Supporting Information

S1 Table Summary of repeat-like unclassified families. (XLSX)

S2 Table Differential Expression of orthologous genes displaying polymorphic TE insertions. (DOCX)

S3 Table Percentage of TE content in 18 fungal species. (XLSX)

S1 Fig TE-mediated loss of conservation between *P*. *ostreatus* and other basidiomycetes on chromosome VII (A = *Coprinopsis cinerea*, B = *Laccaria bicolor*, C = *Phanerochaete chrysosporium*, D = *Postia placenta*, E = *Schizophyllum commune*, F = *Serpula lacrymans*). (PDF)

S2 Fig Phylogenetic reconstruction of Gypsy LTR-retrotransposons. (PDF)

S3 Fig Expression (A) and phylogeny (B) of *P*. *ostreatus* DNA methyltransferases. (TIF)

S1 Dataset Annotation of transposable elements in the PC15 genome. (GFF)

S2 Dataset Annotation of transposable elements in the PC9 genome. (GFF)

S3 Dataset Estimation of PC9 TE content from 454 sequencing reads. (XLSX)

S1 Text Supplementary Methods. (DOCX)
