# Supplementary material for: Transposable Elements versus the Fungal Genome: Impact on Whole-Genome Architecture and Transcriptional Profiles
Source: PLoS Genet. 2016 Jun 13;12(6):e1006108. doi: 10.1371/journal.pgen.1006108 (PMC4905642; doi:10.1371/journal.pgen.1006108)
Supplement: S2 Table — (DOCX) [file pgen.1006108.s002.docx]

| **TE insertions in PC15** | | | | | |
| --- | --- | --- | --- | --- | --- |
| **PC9 (noTE)** | **PC15 (TE)** | **TE family** | **PC9 FPKM** | **PC15 FPKM** | **Description** |
| 120845 | 1099919 | Copia_2 | 13.1 | 0.14 | Unknown function |
| 100052 | 1106124 | Gypsy_3 | 32.6 | 0.49 | Unknown function |
| 86999 | 160984 | Copia_11 | 13.9 | 0.3 | Unknown function |
| 98979 | 154062 | Gypsy_24 | 61.6 | 1.44 | Unknown function |
| 49583 | 166872 | Gypsy_16 | 46.6 | 1.3 | Unknown function |
| 90025 | 158900 | Gypsy_9 | 19.7 | 0.57 | Unknown function |
| 68169 | 1091908 | Gypsy_23 | 1.5 | 0.12 | Zinc finger, C2H2-type |
| 91331 | 1044593 | Gypsy_3 | 1.5 | 0.16 | Glycoside Hydrolase Family 131 protein |
| 89531 | 1097443 | Copia_5 | 142.2 | 15.73 | Unknown function |
| **TE insertions in PC9** | | | | | |
| **PC9 (TE)** | **PC15 (noTE)** | **TE family** | **PC9 FPKM** | **PC15 FPKM** | **Description** |
| 95253 | 167769 | Gypsy_3 | 0.3 | 26.39 | Unknown function |
| 91123 | 154703 | Copia_7 | 8.4 | 147.56 | GMC oxidoreductase |
| 58056 | 1090089 | HELPO1 | 9.4 | 94.15 | Pyridoxamine 5'-phosphate oxidase |
| 81558 | 152288 | Copia_8 | 6.8 | 56.23 | Dimeric alpha-beta barrel |
| 117290 | 176657 | Gypsy_19 | 11.4 | 89.85 | Zinc finger, C2H2-type |
| 125737 | 1085502 | Gypsy_3 | 0.5 | 3.77 | Cytochrome P450 |
| 126274 | 161195 | DIRS_2 | 0.5 | 3.61 | Unknown function |
| 101053 | 30924 | Copia_12 | 31 | 183.32 | NAD(P)-binding |
