# Supplementary figures and images for: Transposable Elements versus the Fungal Genome: Impact on Whole-Genome Architecture and Transcriptional Profiles
Source: PLoS Genet. 2016 Jun 13;12(6):e1006108. doi: 10.1371/journal.pgen.1006108 (PMC4905642; doi:10.1371/journal.pgen.1006108)

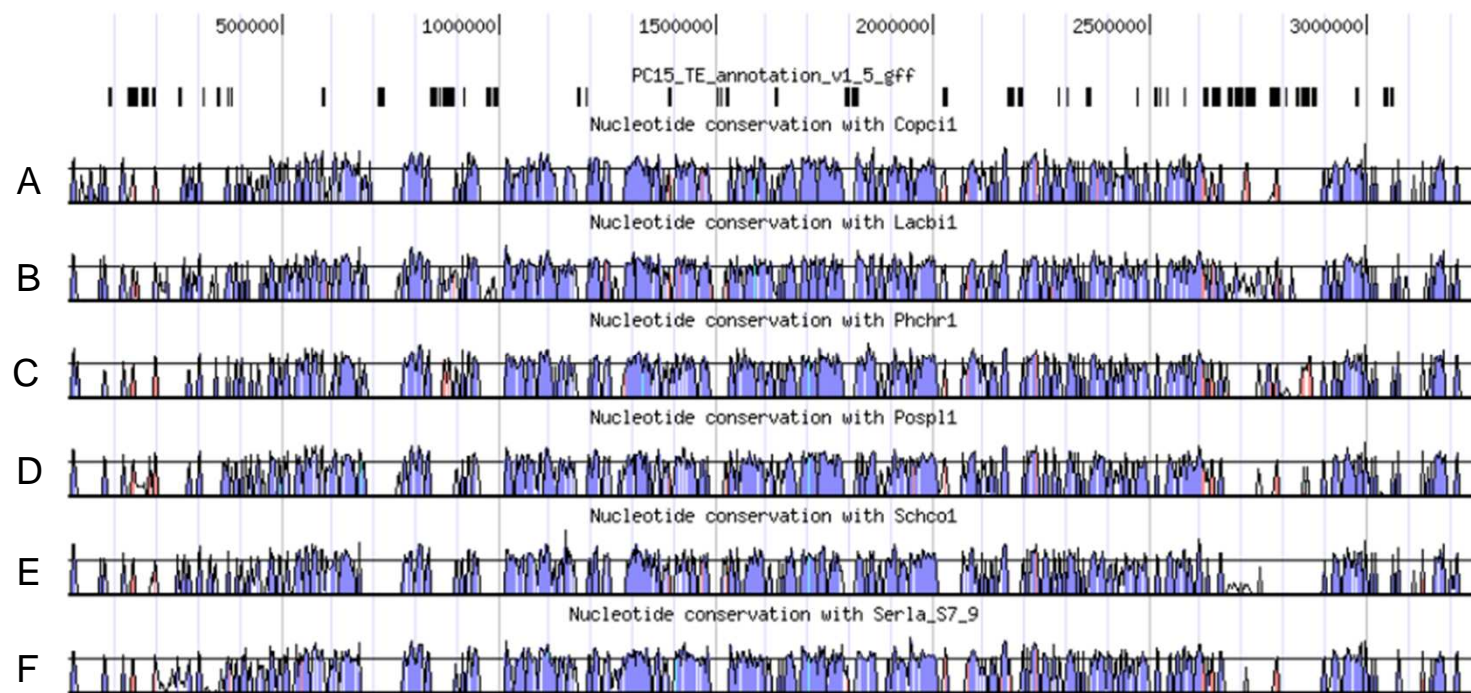

Supplement: S1 Fig — (PDF) [file pgen.1006108.s004.pdf]

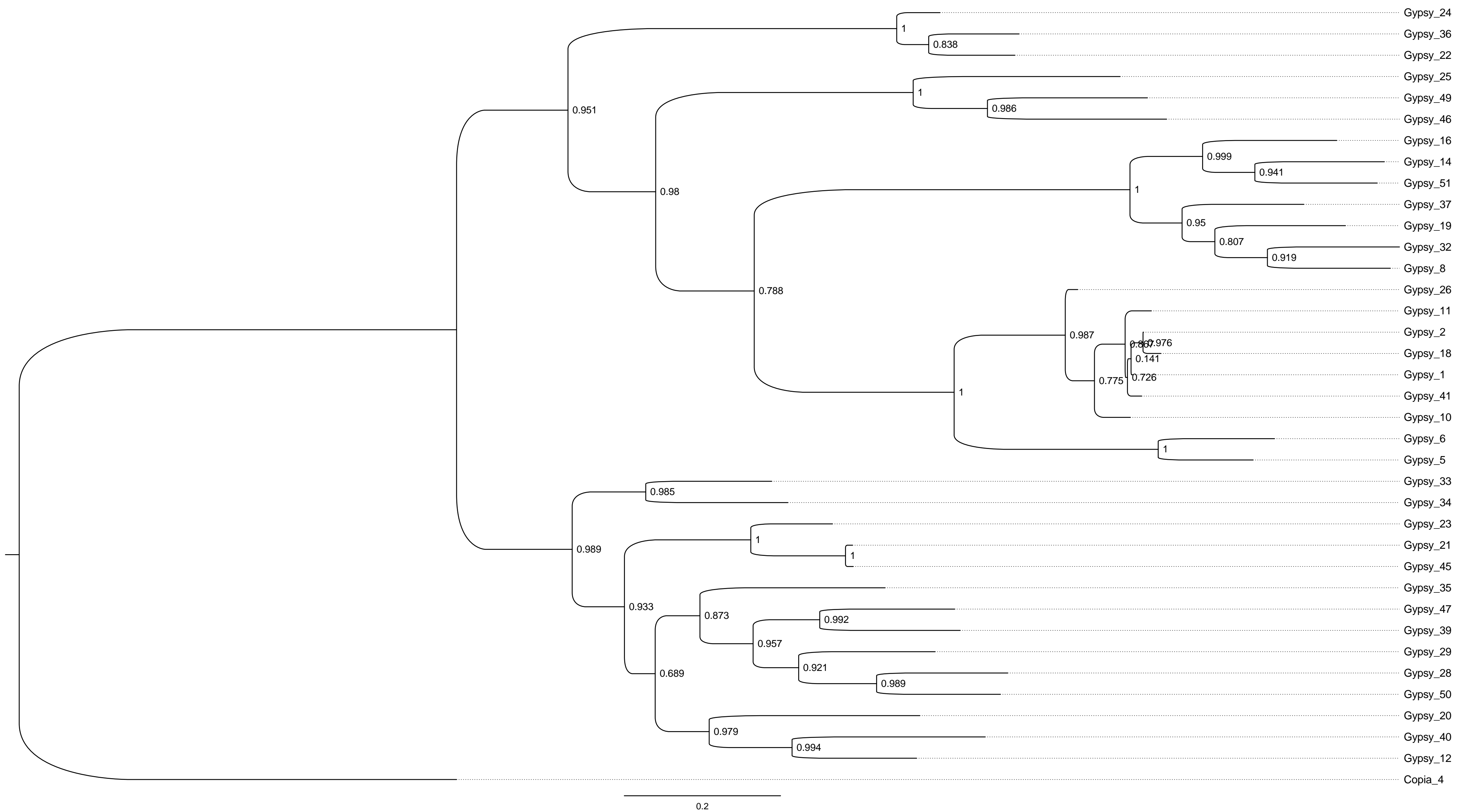

Supplement: S2 Fig — (PDF) [file pgen.1006108.s005.pdf]

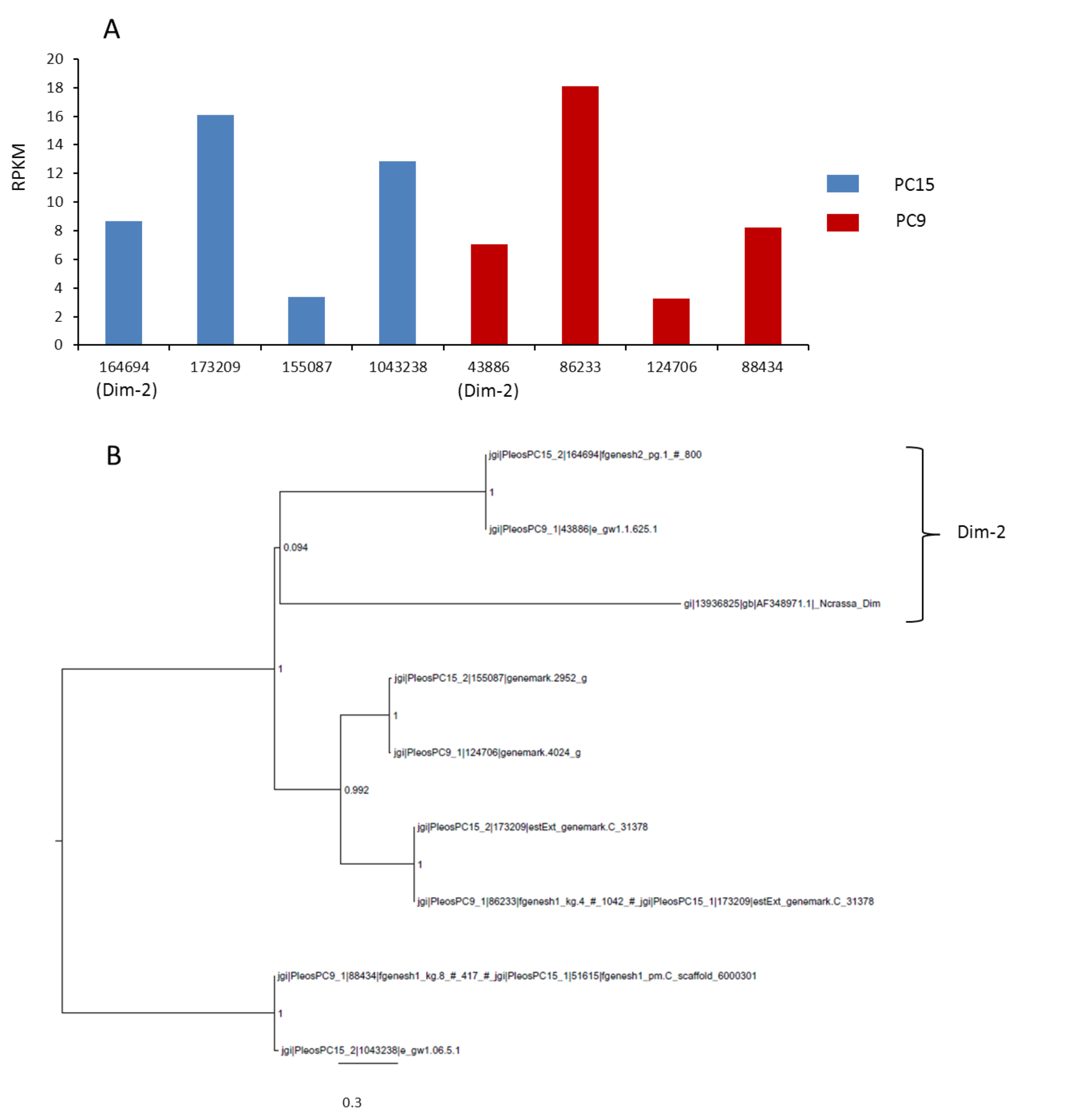

Supplement: S3 Fig — (TIF) [file pgen.1006108.s006.tif]
