# Supplementary material for: Transposable Elements versus the Fungal Genome: Impact on Whole-Genome Architecture and Transcriptional Profiles
Source: PLoS Genet. 2016 Jun 13;12(6):e1006108. doi: 10.1371/journal.pgen.1006108 (PMC4905642; doi:10.1371/journal.pgen.1006108)
Supplement: S1 Text — (DOCX) [file pgen.1006108.s010.docx]

**SUPPLEMENTARY METHODS**

**PCR amplification of polymorphic TE insertions.**

PCR reactions were performed using the following primers:

I-Fw (CGACTCCTCGGTGTCTGATT), I-Rv (ATACCCCAACGACAGTTTGC), II-Fw, (TCCTTTTCGCTGTCTTCCAT), II –Rv (GCACAGGGTCCCTAATCAAA), III-Fw (AGAAGCAGCTGCCTGTCAAC) , III –Rv (TTTTCTTGCTGTTCCGCTTT), IV-Fw(CGCATGGTCGATGTCAATAA), IV-Rv(CGGGTGCCTACGTGTTAAGT), V-Fw (CGACAGCAGTTGCTGGAGTA), V –Rv (TGGCGGTAATAACCAAGGAG), VI-Fw (TGACGGATTAGTTTCGAGCA), VI –Rv (AGGCGTCTGTACCCGATCTA), VII-Fw (TAAGGGTTTGGACCAAGCTG),VII-Rv (CAAGCCCCATTTCATATGCT), VIII-Fw(ATGTTACCTCCGTTGCCTTG),VIII-Rv(AAGACTGCGGTAGGCATTGT).

**Phylogeny of LTR-retrotransposons conserved domains**

Reverse transcriptase and RNAse domains of Gypsy and Copia elements present were extracted from LTR-retrotransposons of the TE library using exonerate [1] and aligned with MUSCLE [2]. The alignments were trimmed using trimAl [3] with the default parameters, and an approximate maximum likelihood tree was constructed using FastTree [4] and edited with Figtree (<http://tree.bio.ed.ac.uk/software/figtree/>).

**Expression and phylogeny of *P. ostreatus* DNA methyltransferases**

Searches were performed in the PC15 and PC9 homepages of the JGI database for retrieving every protein classified under the GO term “DNA methylation”. A protein domain analysis was performed using the Conserved Domain Database [5], and only those carrying the Dcm domain (Site-specific DNA-cytosine methylase, COG0270) were retained. The Dim-2 DNA methyltransferase of Neurospora crassa (gi_28921348) was obtained from the NCBI database. The phylogenetic analysis was carried out using the protein sequences and the same methodology as for LTR-retrotransposons.

**REFERENCES**

1. Slater GS, Birney E. Automated generation of heuristics for biological sequence comparison. Bmc Bioinformatics. 2005;6: 31.

2. Edgar RC. MUSCLE: multiple sequence alignment with high accuracy and high throughput. Nucleic Acids Res. 2004;32: 1792-1797.

3. Capella-Gutierrez S, Silla-Martinez JM, Gabaldon T. trimAl: a tool for automated alignment trimming in large-scale phylogenetic analyses. Bioinformatics. 2009;25: 1972-1973.

4. Price MN, Dehal PS, Arkin AP. FastTree: computing large minimum evolution trees with profiles instead of a distance matrix. Mol Biol Evol. 2009;26: 1641-1650.

5. Marchler-Bauer A, Anderson JB, Chitsaz F, Derbyshire MK, DeWeese-Scott C, Fong JH, Geer LY, Geer RC, Gonzales NR, Gwadz M et al: CDD: specific functional annotation with the Conserved Domain Database. Nucleic Acids Res. 2009;37:D205-D210.
